# CD133 Stimulates Cell Proliferation via the Upregulation of Amphiregulin in Melanoma

**DOI:** 10.3390/cells13090777

**Published:** 2024-05-02

**Authors:** Cynthia M Simbulan-Rosenthal, Nusrat Islam, Yogameenakshi Haribabu, Ryyan Alobaidi, Azadeh Shalamzari, Garrett Graham, Li-Wei Kuo, Peter Sykora, Dean S Rosenthal

**Affiliations:** 1 Department of Biochemistry and Molecular & Cellular Biology, Georgetown University School of Medicine, Washington, DC 20057, USA; simbulac@georgetown.edu (C.M.S.-R.); ni98@georgetown.edu (N.I.); yh577@georgetown.edu (Y.H.); raa125@georgetown.edu (R.A.); afs79@georgetown.edu (A.S.); gtg9@georgetown.edu (G.G.); lk702@georgetown.edu (L.-W.K.); 2 Amelia Technologies, LLC., Washington, DC 20001, USA; peters@ameliatechnologies.com

**Keywords:** melanoma initiating cells, melanoma stem cells, CD133, AREG, EGFR, MAPK pathway, cell proliferation

## Abstract

CD133, a cancer stem cell (CSC) marker in tumors, including melanoma, is associated with tumor recurrence, chemoresistance, and metastasis. Patient-derived melanoma cell lines were transduced with a Tet-on vector expressing CD133, generating doxycycline (Dox)-inducible cell lines. Cells were exposed to Dox for 24 h to induce CD133 expression, followed by RNA-seq and bioinformatic analyses, revealing genes and pathways that are significantly up- or downregulated by CD133. The most significantly upregulated gene after CD133 was amphiregulin (*AREG*), validated by qRT-PCR and immunoblot analyses. Induced CD133 expression significantly increased cell growth, percentage of cells in S-phase, BrdU incorporation into nascent DNA, and PCNA levels, indicating that CD133 stimulates cell proliferation. CD133 induction also activated EGFR and the MAPK pathway. Potential mechanisms highlighting the role(s) of CD133 and AREG in melanoma CSC were further delineated using AREG/EGFR inhibitors or siRNA knockdown of *AREG* mRNA. Treatment with the EGFR inhibitor gefitinib blocked CD133-induced cell growth increase and MAPK pathway activation. Importantly, siRNA knockdown of AREG reversed the stimulatory effects of CD133 on cell growth, indicating that AREG mediates the effects of CD133 on cell proliferation, thus serving as an attractive target for novel combinatorial therapeutics in melanoma and cancers with overexpression of both CD133 and AREG.

## 1. Introduction

Malignant melanoma (MM) remains the most lethal form of skin cancer and the fourth-most common cancer in the US, with 98,000 diagnoses and 8000 deaths in 2023 [1]. Worldwide, 325,000 people were diagnosed in 2020, representing more than 20% of all cancers, with 57,000 deaths; these rates may rise to 510,000 diagnoses and 96,000 deaths by 2040 [2]. While mortality has recently declined with improved disease management, the incidence has remained high, partly as a result of aging populations, with increased numbers of mutations, although tanning beds and solar UV exposure contribute to high rates of MM in younger individuals [1].

Melanomagenesis is associated with driver mutations in the RAS-RAF-MEK-ERK mitogen activated protein kinase (MAPK) cell signaling pathway, including *BRAF^V600^* [3], *NRAS^Q61^* [4], *CKIT*, *NF1*. *BRAF* mutations activate the MAPK pathway, while the other three function upstream and induce both MAPK and PI3K/AKT pathways, making them more problematic [5,6]. Therefore, while *BRAF^V600^*-mutant melanoma shows improved clinical outcomes with targeted inhibitors of BRAF (e.g., vemurafenib, dabrafenib, encorafenib) and MEK (e.g., trametinib, cobimetinib, binimetinib), *NRAS*-mutant MM respond poorly [7], and resistance develops rapidly, even when used in combination with immune checkpoint inhibitors [8], with a majority of patients likely to die within a year of their disease. Hence, new therapy options are needed, particularly for those with *NRAS*-mutant melanoma. 

Resistance to current therapies and tumor recurrence is thought to be the result of subpopulations of melanoma-initiating cells (MIC) or melanoma stem cells (MSC) that are capable of self-renewal and potency and are implicated in tumor initiation, progression, and the generation of phenotypic heterogeneity. CD133, a glycosylated penta-span transmembrane protein, is a putative stem cell marker detected in stem cells in normal renewing tissues [9], as well as the CSC of the brain [10], ovary [11], liver [12], prostate [13], pancreas [14], and colon [15]. Our earlier studies strongly correlated CD133 immunopositivity with tumor progression and recurrence in cutaneous melanoma [16]. The mechanisms by which CD133 controls melanoma progression thus remain to be explored, especially if it activates multiple upstream pathways that make MM more difficult to treat. 

We previously investigated the roles of CD133 in invasion and metastasis, as well as in resistance to targeted MAPK inhibitors (MAPKi). Following cell sorting and conditional reprogramming, CD133(+) cells exhibited higher invasion and metastasis, which is reversed by siRNA and CRISPR-Cas9 knockdown of CD133 in several patient-derived melanoma cell lines [17]. Increased cell survival and drug resistance in CD133(+) MIC cells, compared to CD133(-) cells, suggested that CD133 confers resistance to MAPKi, trametinib and dabrafenib. siRNA knockdown of CD133 re-sensitized cells to both MAPKi, indicating a causal relationship between CD133 and drug resistance in melanoma [18]. We further examined potential molecular mechanisms by which CD133 increases cell survival and resistance in response to trametinib. While CD133-overexpressing MIC cells displayed increased cell viability and a reduced apoptotic response to trametinib, CRISPR-Cas9 knockout of CD133 reversed this phenotype in different melanoma cell lines, re-sensitizing cells to trametinib. Doxycycline (Dox)-inducible expression of CD133, on the other hand, diminished trametinib-induced apoptosis and upregulated anti-apoptotic pAKT and BCL-2 family proteins, suggesting that CD133 increases drug resistance against current targeted therapies through activation of an AKT- p-BAD -BCL-2-mediated survival pathway [19]. 

In the current study, we performed RNA-seq and bioinformatic analyses to identify additional genes and pathways that are up- or downregulated as a result of Dox-inducible CD133 expression in melanoma cells that could reveal actionable targets for drug-resistant MM. Interestingly, CD133 expression induced an 8-fold increase in AREG, a 43-kDa transmembrane glycoprotein ligand that binds to the epidermal growth factor receptor (EGFR), strongly activating both the MAPK RAS-RAF-MEK/ERK and PI3K/AKT signaling pathways implicated in cell proliferation, migration, and survival. We further investigated the effects of induced CD133 expression and consequent AREG upregulation on cell proliferation and cell growth in patient-derived human melanoma cells and tested the feasibility of targeting *AREG* by siRNA and eventually small molecule or antibody-based inhibitors.

## 2. Materials and Methods

### 2.1. Cells

Patient-derived de-linked human melanoma cell lines harboring recalcitrant *NRAS* mutations, including BAKP (*NRAS^Q61K^*) and POT (*NRAS^Q61R^*), were maintained in Iscove’s Modified Dulbecco’s Medium (IMDM) supplemented with 10% FBS and 1% penicillin- streptomycin in a 37 °C 5% CO_2_ incubator. Cells were assessed for melanoma markers MART1 and S100 by flow cytometry, and their *BRAF* or *NRAS* mutations were verified by Sanger sequencing as previously described [17].

### 2.2. Generation of Dox-Inducible Cells 

We used the Dox-inducible Tet-On system, a widely-used method for functional studies on the effects of activation of gene expression that is based on the bacterial Tet operon modified for eukaryotic cells. The system serves as a genetic switch that allows reversible control of gene expression, turning on the expression of specific genes with the addition of exogenous Dox, a tetracycline derivative [20,21,22]. To generate Dox-inducible lentivirus that can increase CD133 expression, HEK293FT packaging cells were co-transfected with pLenti-CMV-rtTA3 Blast (Addgene, Watertown, MA, USA) and psPAX2 and pMD2.G (5 µg each; Addgene), with or without pLV-EGFP/Neo-TRE3G-CD133 (10 µg; VectorBuilder Inc., Chicago, IL, USA) using Lipofectamine LTX (ThermoFisher Sci, Waltham, MA, USA). The vector map and detailed sequence of the pLV-EGFP/Neo-TRE3G-CD133 lentiviral vector are shown in Supplementary Materials (Figure S1); detailed information on pLenti-CMV-rtTA3 Blast can be accessed at https://www.addgene.org/26429/sequences/, accessed on 30 April 2024. Medium was replaced after 16 h, and cells were incubated for 48 h to generate lentivirus. Melanoma cells were then transduced by adding viral supernatants (MOI = 1). After 24 h, transduced cells were selected with blasticidin (40 µg/mL) and geneticin (1 mg/mL) for 10 days, as described [17]. 

### 2.3. RNA-seq Analysis

Cells were seeded in 100 mm plates in triplicates and incubated with or without Dox (1 µg/mL) for 24 h. Total RNA, extracted with Trizol reagent (GIBCO BRL, Grand Island, NY), was then subjected to RNA-Seq sequencing to a depth greater than 50 million reads (Genewiz, South Plainfield, NJ, USA). Reads were aligned to the human genome GRCh38 using the STAR aligner (2.7.4a) with GENCODE (v21) annotation. Only significant differences between −Dox and +Dox cells that were reproducible in triplicate and represented a change of 2-fold or greater were considered. Gene-specific analysis (GSA) with Partek^®^ Flow^®^ software was used to identify differentially expressed genes (DEGs) with a threshold FDR value < 0.05 and fold change >±2. These genes were defined as significantly downregulated or upregulated by CD133. 

### 2.4. Pathway Analysis

Gene expression profiles obtained by RNA-Seq analysis were analyzed using Ingenuity, KEGG, and REACTOME pathway enrichment to determine significantly enriched pathways and cellular processes. Functional enrichment analysis and prediction of gene function were performed using Gene Set Enrichment Analysis (http://www.gsea-msigdb.org/gsea/index.jsp, accessed on 30 April 2024). Pathways with a FDR ≤ 0.05 were considered significant.

### 2.5. Quantitative Reverse-Transcription PCR (qRT-PCR)

Total RNA was purified from cell pellets with Trizol Reagent (Gibco BRL, Grand Island, NY) and subjected to qRT–PCR by standard protocols using two-step reverse transcription–PCR (Invitrogen), RNA (1 µg), and specific primers listed below. cDNA was synthesized using a Verso cDNA synthesis kit (ThermoFisher Sci, Waltham, MA, USA).

*CD133* forward—5′-CCC GGG GCT GCT GTT TAT A

*CD133* reverse—5′-ATC ACC AAC AGG GAG ATT G

*AREG* forward—5′-GGT GCT GTC GCT CTT GAT AC

*AREG* reverse—5′-TTC ACG CTT CCC AGA GTA GG

### 2.6. Immunoblot Analysis

Total cell lysates were subjected to SDS-Page electrophoresis, and proteins were transferred to nitrocellulose membranes according to standard procedures. To verify the transfer of proteins and equal loading, membranes were stained with Ponceau S (0.1%). Membranes were then incubated with antibodies to CD133 (Miltenyi Biotec, Auburn, CA, USA), AREG (Santa Cruz Biotech, Dallas, TX, USA), p-EGFR, p-MEK, p-BAD (BioLegend, San Diego, CA, USA), p-ERK, total MEK, ERK, BAD, proliferating cell nuclear antigen (PCNA), cyclin D1, or β-actin (ProteinTech, Rosemont, IL, USA) as loading control. After stripping membranes of antibodies, immunoblots were sequentially re-probed with other antibodies, and immune complexes were detected by incubation with horseradish peroxidase-conjugated antibodies to mouse or rabbit IgG (1:3000), followed by enhanced chemiluminescence (ECL; Pierce, Rockford, IL, USA) and imaging in a GE Healthcare Amersham Imager 600. 

### 2.7. Cell Cycle and Cell Growth Analysis

1.5 × 10^5^ cells were seeded in 6-well plates in triplicates and incubated with 1 µg/mL Dox for 24 h to induce CD133 expression. Cell viability and apoptosis assays have verified that 1 μg/mL Dox for up to 72 h is not cytotoxic for the melanoma cell lines used in the current study [19]. For cell growth assays, total numbers of GFP-expressing cells were imaged and counted in triplicate wells in 3 random microscope fields per well (*n* = 9) daily for 5 days, using an Olympus immunofluorescence microscope equipped with a DP75 digital camera and cellSens imaging software (Evident, Tokyo, Japan). For cell cycle analysis, at the indicated times, cells were collected and fixed in 95% ethanol. Nuclei were stained with propidium iodide (PI) and subjected to flow cytometric analysis for cell cycle analysis/DNA content in a Becton-Dickinson FacStar Plus at the GUMC Flow Cytometry Core Facility. 

### 2.8. Bromodeoxyuridine (BrdU) Staining and Fluorescence Microscopy

To detect BrdU incorporation into newly synthesized DNA, cells were pulse-labeled with 10 µM BrdU for 1 h and fixed in ice-cold 95% ethanol for 30 min, as previously described [23]. Briefly, genomic DNA was denatured with 0.07 M NaOH in 70% ethanol, then washed with PBS, and incubated in a humid chamber for 4 h with antibodies to CD133 (Miltenyi Biotec, Auburn, CA, USA) and BrdU (Cell Signaling, Danvers, MA, USA), followed by secondary antibodies anti-rabbit Alexa Fluor 488 and anti-mouse Alexa Fluor 594, respectively. Cells were then imaged using TRITC filters for BrdU, FITC for CD133, and DAPI for nuclear staining using an Olympus immunofluorescence microscope with a DP75 digital camera and cellSens imaging software (Evident, Tokyo, Japan). 

### 2.9. Statistical Analysis

All statistical analyses were performed using GraphPad Prism9 (GraphPad, San Diego, CA, United States). Experiments were performed in biological triplicates; representative data from three independent experiments are presented. Standardized two-tailed student *t*-tests were used with two group comparisons between control and test samples; *p*-values were obtained to determine significance. Error bars shown on graphs are ±SEM. *p* < 0.05 was considered significant, and *p* < 0.05, *p* < 0.01, *p* < 0.001, or *p* < 0.0001 are denoted with one to four asterisks. 

## 3. Results

### 3.1. RNA-seq Analysis Reveal AREG as the Most Differentially Expressed Gene in Patient-Derived BAKP Melanoma Cells after Doxycycline (Dox)-Inducible Expression of CD133

**Figure 1:**
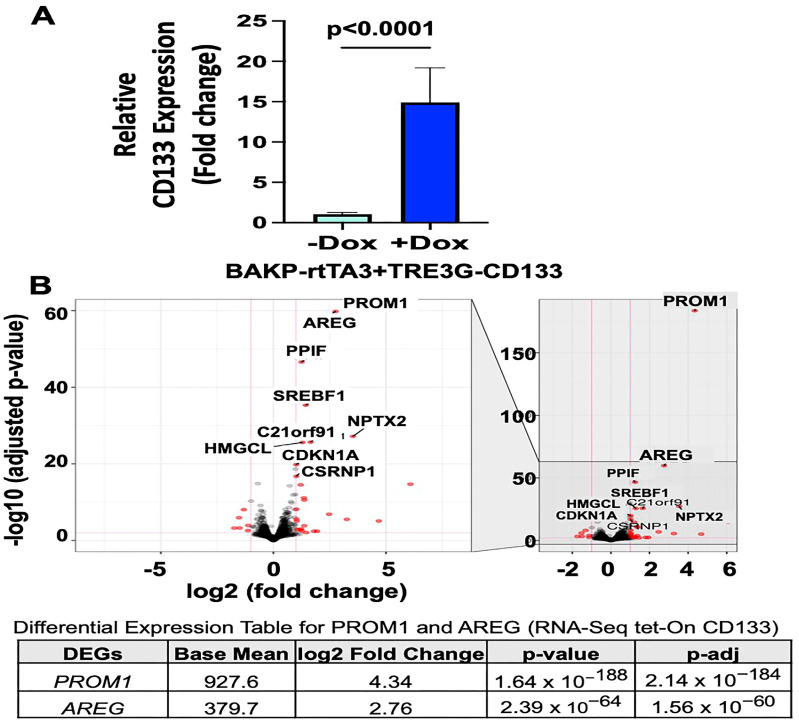
(**A**) qRT-PCR analysis of triplicate RNA samples from Dox-induced vs. uninduced BAKP cells were subjected to RNA-seq analysis. (**B**) Volcano plot of RNA-seq analysis shows top upregulated genes (top panel), while differential expression table shows fold increase in *PROM1* and *AREG* expression (bottom panel). An adjusted *p* value (q-value < 0.05) and fold change (log2 fold change ≥ ±2) were used to identify significantly up- or downregulated genes. The top significantly upregulated genes (FDR < 0.01) are shown in the volcano plot.

To characterize and target CD133-positive cancer stem cells, the patient-derived de-identified melanoma cell line, BAK parental (BAKP), harboring a refractory *NRAS* driver mutation and expressing low basal CD133 levels, was transduced with a Tet activator (rtTA3, Addgene) and a Tet-on vector expressing CD133 (TRE3G-CD133, VectorBuilder). After selection, stable cell lines were treated with Dox (1 µg/mL) for 24 h to induce CD133 expression. mRNA was extracted from biological triplicates and subjected to qRT-PCR analysis, which confirmed a robust 15-fold increase in CD133 expression in Dox-induced compared to control BAKP cells (Figure 1A).

Differentially expressed genes (DEGs) regulated by CD133 were then identified by subjecting the RNA samples to RNA-seq analysis, followed by gene-specific analysis (GSA) with Partek^®^ Flow^®^ software. Genes with a threshold FDR value < 0.05, and fold change of >±2 were defined as significantly up- or downregulated by CD133. The volcano plot showed that the topmost significantly upregulated gene (FDR < 0.01), after *CD133* (*PROM1*), was *AREG* (Figure 1B). Dox induction increased CD133 expression ~16-fold, resulting in an ~8-fold upregulation of *AREG* in BAKP melanoma cells. 

### 3.2. Validation of Differentially Expressed Genes in RNA-seq by Immunoblot Analysis in BAKP Melanoma Cells after Dox-Inducible Expression of CD133

**Figure 2:**
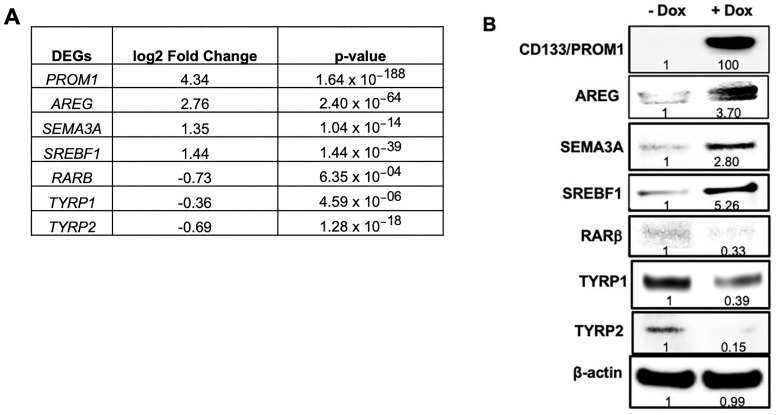
Validation of RNA-seq data (**A**) by immunoblot analysis (**B**) showing upregulation of CD133, AREG, SEMA3A, and SREBF1, and downregulation of differentiation genes *RARB*, *TYRP1*, and *TYRP2* in Dox-induced BAKP cells. Cells were seeded in 100 mm plates, incubated with or without 1 µg/mL Dox for 24 h, and subjected to immunoblot analysis with antibodies to CD133. Immunoblots were stripped of antibodies and re-probed with antibodies to AREG, SEMA3A, SREBF1, RARB, TYRP1, TYRP2, and β-actin for loading control. After normalizing to β-actin, densitometric analysis comparing intensities of protein bands relative to bands with the highest intensities, is shown in immunoblots.

Differential expression of several genes that were significantly altered by induced CD133 expression in the RNA-seq data (Figure 2A) was verified by immunoblot analysis (Figure 2B), including the proteins AREG, semaphorin 3A (SEMA3A), sterol regulatory element-binding transcription factor 1 (SREBF1), tyrosinase-related protein 1 (TYRP1), TYRP2, and retinoic acid receptor b (RARB). In agreement with the RNA-seq results, immunoblot analysis revealed marked upregulation of AREG, SEMA3A, and SREBF1 and downregulation of RARB, TYRP1, and TYRP2 in CD133-expressing BAKP cells (Figure 2B). CD133 upregulated SEMA3A and SREBF1 by 3- and 5-fold, respectively; these genes have been implicated in the tumor progression of different cancers [24,25,26]. SREBF1, also known as SREBP1, is a transcription factor regulating lipogenesis and is involved in the metabolic reprogramming of various cancers [27,28,29]. TYRP1 and TYRP2, both melanocyte differentiation markers and key enzymes for melanin synthesis, were significantly downregulated in CD133-expressing BAKP cells (Figure 2B), consistent with our RNAseq data. Likewise, RARB, implicated in cell signaling in embryogenesis and cell differentiation, was also downregulated by CD133. Inhibition of differentiation during the progression of CD133-expressing melanoma stem cells may therefore be mediated by downregulation of RARB, TYRP1, and TYRP2, among other mechanisms. 

### 3.3. Pathway Analysis of Significantly Altered Genes in BAKP Melanoma Cells after Dox-Induced Expression of CD133

**Figure 3:**
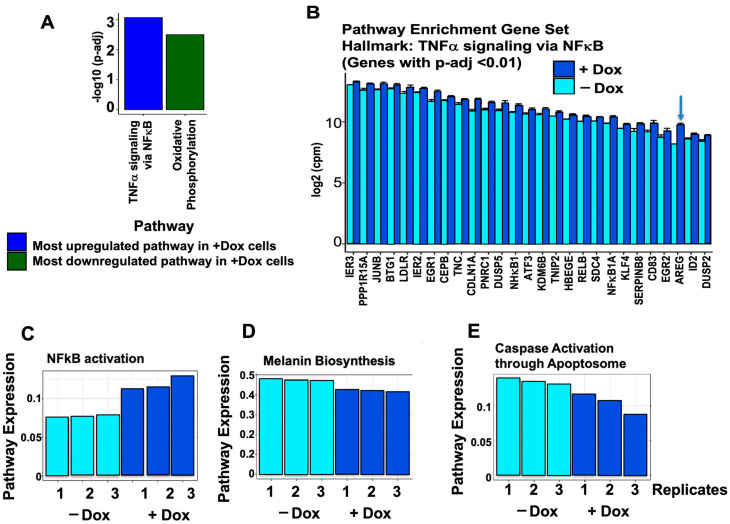
Reactome pathway analysis of DEGs from RNA-seq analysis of Dox-induced vs. uninduced BAKP cells. (**A**) Most significantly upregulated and downregulated pathway. (**B**) Most significantly upregulated DEGs included in the “TNFα signaling via NF-κB” pathway. Blue arrow indicates AREG. (**C**–**E**) Expression of NF-κB pathway (**C**) is upregulated, while pathway expression for melanin biosynthesis (**D**) and caspase activation via the apoptosome (**E**) are both downregulated in CD133-expressing BAKP melanoma cells.

Reactome Pathway Analysis on DEGs was next used to identify significant relevant pathways, interactions, and associated networks altered by Dox-induced CD133 expression. When the lists of significantly up- and downregulated genes from RNA-seq data were analyzed by pathway analysis, “Tumor necrosis factor a (TNFα) signaling via Nuclear factor kB (NF-κB)” was ranked as the most significantly upregulated pathway, whereas “Oxidative Phosphorylation” was the most significantly downregulated (Figure 3A). Downregulation of genes in the oxidative phosphorylation pathway implies a switch to oxidative glycolysis as the main energy-yielding pathway in melanoma stem cells, as seen in most tumors (Warburg effect). Interestingly, DEGs in the most upregulated pathway, “TNFα signaling via NF-κB,” consist of genes involved in cell cycle regulation and cell proliferation, including AREG (Figure 3B). These genes may mediate the cell cycle dysregulation, increased cell proliferation, and reprogramming in CD133-expressing melanoma stem cells. Upregulation and activation of NF-κB contribute to unregulated cell proliferation in cancer cells, given that NF-κB transcription factors regulate the expression of cell cycle regulators such as cyclin A, cyclin D1, or cyclin-dependent kinase 6 (CDK6). The stemness gene Kruppel-like factor 4 (*KLF4*) is also upregulated by CD133 in this NF-κB pathway (Figure 3B), in agreement with our previous results showing upregulation of other stemness genes such as *Oct4* and *ABCB5* in CD133(+)-enriched BAKP melanoma stem cells [18]. As expected, the reactome analysis revealed that the NF-κB activation pathway is upregulated with CD133 expression in these cells (Figure 3C). 

Consistent with downregulation of the melanocyte differentiation markers and key enzymes for melanin synthesis, TYRP1 and TYRP2, (Figure 2), pathway expression for the melanin biosynthesis pathway was markedly downregulated in CD133-expressing BAKP melanoma cells (Figure 3D). Decreased differentiation is in keeping with the increased cell proliferation signals induced by CD133. Finally, caspase activation via the apoptosome, central to the mitochondrial-mediated pathway of apoptosis, was similarly downregulated by CD133 expression (Figure 3E), in line with a reduced apoptotic response to targeted therapies and increased drug resistance in CD133-expressing MICs [18,19]. 

### 3.4. Both the Intracellular AREG Precursor and Secreted Forms of AREG Are Upregulated in BAKP Melanoma Cells after Dox-Induced CD133 Expression

**Figure 4:**
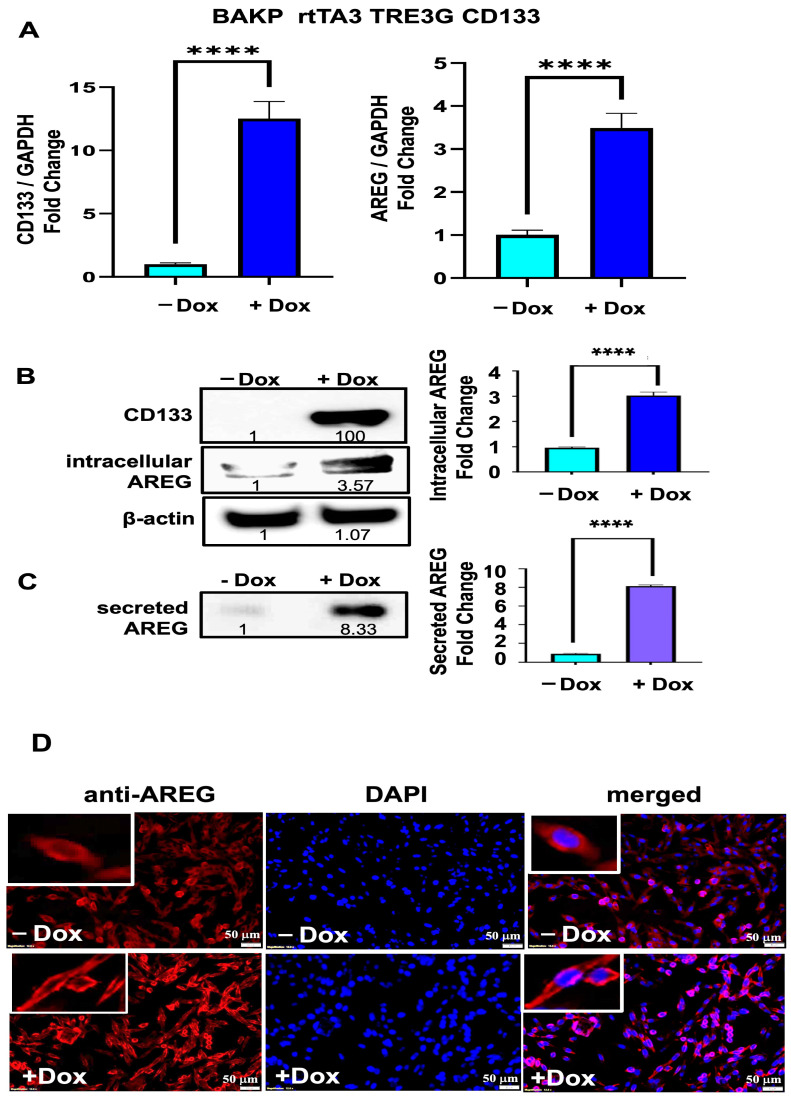
(**A**) Upregulated expression of *CD133* (**left** panel) and *AREG* (**right** panel) mRNA in Dox-induced BAKP cells was verified by qRT-PCR analysis. Cells were incubated with or without 1 µg/mL Dox for 24 h; total RNA was extracted and subjected to qPCR. Results shown are the means ± SEM of three replicates of a representative experiment; essentially the same results were obtained in three independent experiments. *p* < 0.05 was considered significant; **** *p* < 0.0001. (**B**) CD133 and intracellular AREG precursor protein levels were compared between control and Dox-induced BAKP cells by immunoblot analysis of total cell lysates with antibodies to CD133; membranes were stripped and re-probed with antibodies to AREG and β-actin as loading control. Densitometric analysis is shown in immunoblots, comparing intensities of protein bands relative to those of control -Dox, after normalizing to β-actin. (**C**) Cell culture media was collected, concentrated and subjected to immunoblot analysis with antibodies to AREG to detect secreted ligand forms of AREG. (**D**) AREG localization in the cytoplasm and cell membrane, not in the nucleus, is verified by immunofluorescence staining with antibodies to AREG. Representative images of BAKP cells uninduced (-Dox, upper panel) or induced with Dox (+Dox; lower panel) for 24 h, fixed, and subjected to immunofluorescence staining with antibodies to AREG, followed by DAPI for nuclear staining.

Due to the robust upregulation of AREG and its role in the tumor progression of different cancer types, which highlights its suitability as a therapeutic target, we further examined whether AREG mediates the effects of induced CD133 expression in melanoma stem cells. Dox-inducible BAKP cells were exposed to Dox for 24 h, followed by RNA extraction and qRT-PCR analysis. Consistent with RNA-seq results (Figure 1), CD133 expression increased 13-fold, inducing a 3.5-fold increase in AREG RNA in +Dox compared to −Dox cells, as assessed by qRT-PCR analysis (Figure 4A).

Immunoblot analysis of total cell lysates further revealed a 3.6-fold increase in the full-length AREG transmembrane precursor protein in +Dox relative to −Dox cells, coincident with induced CD133 expression in +Dox cells (Figure 4B). Since AREG precursor protein is released extracellularly as soluble AREG ligand after cleavage by metalloprotease TACE/ADAM17 [30], we next determined if AREG TM precursor is likewise secreted as AREG ligand by BAKP melanoma cells. Culture media were collected in the same experiment, concentrated with centrifugal filters (Amicon), and analyzed by immunoblot analysis with antibodies to AREG. Interestingly, the concentrations of the secreted ligand form of AREG in the medium of CD133-expressing +Dox cells were 8-fold higher than Dox cells (Figure 4C). Immunofluorescence analysis further reveals that AREG is localized in the cytoplasm and cell membrane and not in the nuclei of melanoma cells. Consistent with immunoblot results, Dox-induced CD133-expressing cells exhibit higher intracellular AREG levels (Figure 4D); CD133 does not cause relocalization into the nucleus. CD133 may therefore exert both autocrine, juxtracrine, and paracrine stimulation of nearby cells through AREG expression at the cell membrane and via secretion. 

### 3.5. Upregulation of Intrcellular AREG Precursor and Secreted Forms of AREG Is Reproduced in Another Melanoma Cell Line (POT) after Dox-Inducible Expression of CD133

**Figure 5:**
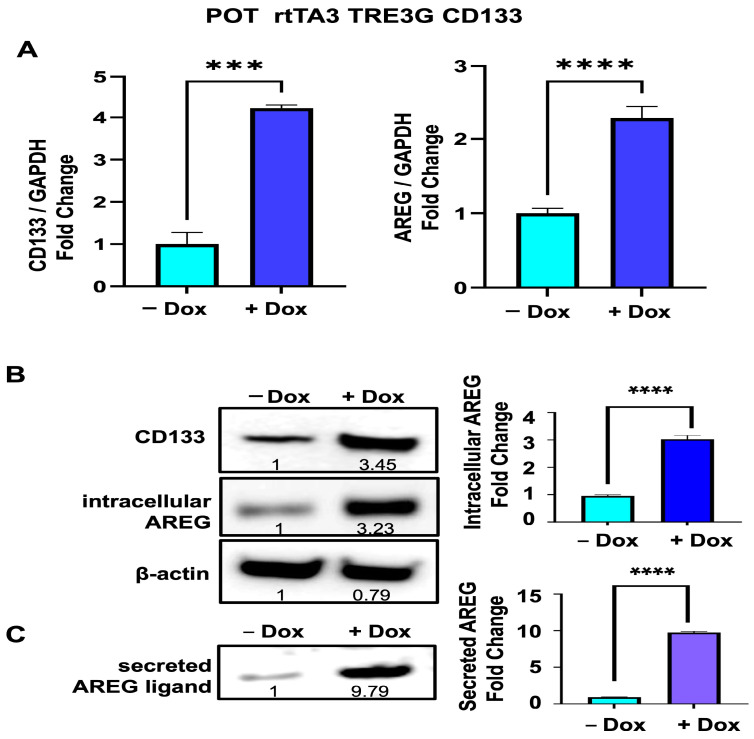
(**A**) Upregulated expression of *CD133* (**left** panel) and *AREG* (**right** panel) in Dox-induced POT cells (+Dox), as verified by qRT-PCR analysis. Results shown are the means ± SEM of three replicates of a representative experiment; essentially the same results were obtained in three independent experiments. *p* < 0.05 was considered significant; ***, **** represent *p* < 0.001 and *p* < 0.0001, respectively. (**B**) CD133 and intracellular AREG precursor protein levels were compared between control and Dox-induced POT cells by immunoblot analysis of total cell extracts with antibodies to CD133; membranes were stripped and re-probed with antibodies to AREG and β-actin as loading control. (**C**) Cell culture media was collected, concentrated and subjected to immunoblot analysis with anti-AREG to detect secreted ligand forms of AREG. Densitometric analysis is shown in immunoblots, comparing intensities of protein bands relative to bands with the highest intensity, after normalizing to β-actin.

To determine if CD133 also upregulates *AREG* expression in other melanoma cell lines, we established another Dox-inducible cell line derived from a second patient-derived melanoma (POT), harboring an *NRAS^Q61R^* mutation. While POT cells exhibit higher basal CD133 levels compared to BAKP cells, CD133 expression was further enhanced in Dox-inducible POT cells by incubation with Dox for 24 h. qRT-PCR analysis revealed that, similar to BAKP cells, following Dox induction, *CD133* expression was significantly upregulated 4-fold in POT cells, concomitant with a >2-fold increase in *AREG* RNA relative to uninduced cells (Figure 5A). 

Cell extracts derived from Dox-induced and uninduced POT cells were next analyzed by immunoblot analysis to determine CD133 protein levels and intracellular TM precursor protein levels of AREG. As with BAKP cells, a robust 3-fold increase in CD133 protein levels resulted in a similar 3-fold increase in intracellular AREG in +Dox vs. −Dox cells (Figure 5B). Culture media was then collected and concentrated to assess levels of secreted ligand forms of AREG by immunoblot analysis. In response to Dox induction, POT cells secreted an 8-fold greater concentration of the soluble AREG ligand form (Figure 5C). AREG RNA and protein levels in both BAKP and POT melanoma cells are therefore markedly elevated in response to CD133 upregulation, resulting in enhanced levels of the transmembrane precursor as well as the secreted mature ligand forms of AREG.

### 3.6. Dox-Induced CD133 Expression Increases the Percent of Cells in the S-Phase of the Cell Cycle, Leading to Increased Cell Growth in BAKP Melanoma Cells

**Figure 6:**
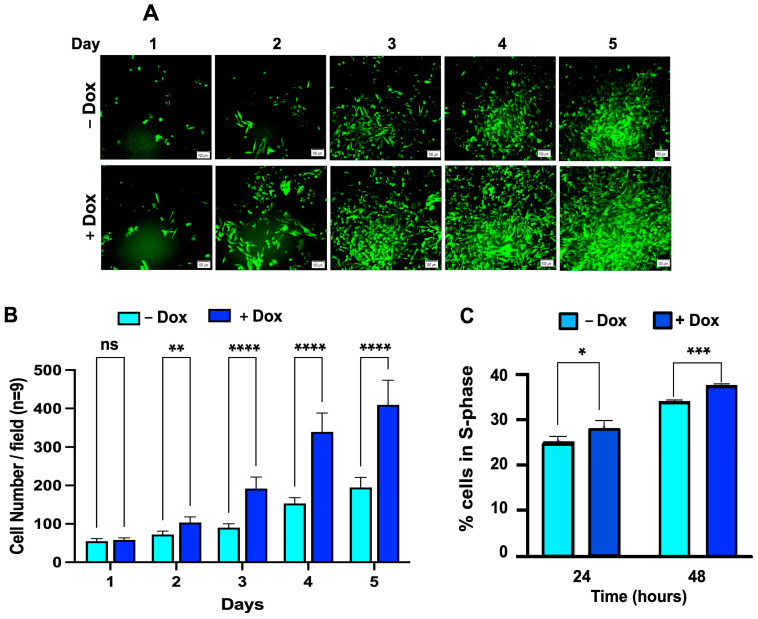
CD133-expressing Dox-induced BAKP cells exhibit increased (**A**,**B**) cell growth, and (**C**) percentage of cells in S-phase, compared to uninduced BAKP cells. Cells were seeded in equal numbers in 6-well plates in triplicates, and incubated for 24 h with 1 µg/mL Dox to induce CD133 expression. Cell growth assays: (**A**,**B**), GFP-expressing BAKP cells were seeded in equal numbers in 6-well plates +/− Dox, and then imaged and counted daily for 5 days in triplicate wells, in 3 random microscope fields per well (*n* = 9); representative images of cells (**A**) and cell counts (**B**) over 5 days. (**C**) Cell cycle analysis: cells were collected at indicated times, fixed in 95% ethanol, stained with PI, and the percentage (%) of cells in S-phase of the cell cycle was quantified by flow cytometric analysis. Results shown are the means ± SEM of three replicates of a representative experiment; essentially the same results were obtained in three independent experiments. *p* < 0.05 was considered significant; *, **, ***, **** represent *p* < 0.05, *p* < 0.01, and *p* < 0.001, and *p* < 0.0001, respectively.

AREG is involved in cell proliferation and migration via its binding to and activation of the epidermal growth factor receptor (EGFR), which then activates the MAPK and PI3K/AKT signaling pathways. To examine the effects of CD133-mediated upregulation and secretion of AREG on cell proliferation, we performed cell growth assays on BAKP cells in the presence or absence of Dox. GFP-expressing BAKP cells were seeded in equal numbers in 6-well plates, incubated with or without Dox, and then imaged and counted daily for 5 days (*n* = 9), as described in Materials and Methods. Dox-induced CD133-expressing BAKP cells exhibited significantly higher total cell numbers at all time points after day 1 (Figure 6A,B). 

Higher cell numbers may be due to either stimulation of cell proliferation, cell survival, or both. To determine if the elevated cell numbers are attributable to increased cell proliferation, CD133 expression was induced in cells by incubation with Dox for 24 h, then analyzed by cell cycle analysis at indicated time points. Consistent with cell growth assays, a significant increase in the percentage of proliferative cells in the S-phase of the cell cycle was noted in BAKP cells induced to express CD133 (Figure 6C). CD133 therefore increases rates of melanoma cell growth, at least in part by enhancing cell proliferation.

### 3.7. Enhanced Cell Proliferation in Dox-Induced CD133-Expressing BAKP Cells, as Shown by BrdU Incorporation into Newly Synthesized DNA

**Figure 7:**
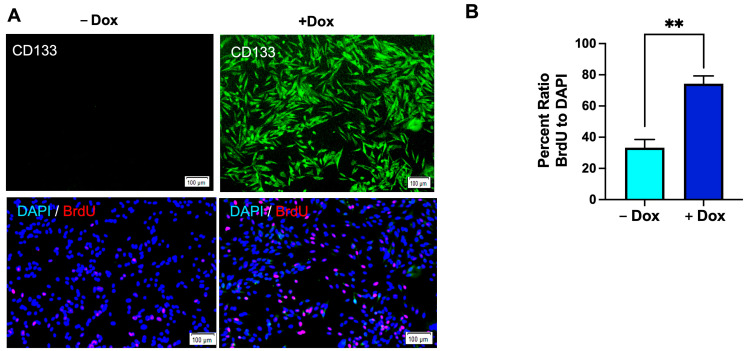
CD133-expressing Dox-induced BAKP cells exhibit increased DNA replication, as assesed by BrdU incorporation into newly synthesized DNA. Cells were induced with Dox for 24 h, synchronize in the cell cycle by serum starvation for 48 h, and stimulated to proliferate and reenter S-phase by serum addition. Cells were pulsed with BrdU 24 h after release into the S-phase, and subjected to immunofluorescence imaging using antibodies specific for BrdU and CD133. (**A**) Representative images of CD133-expressing cells (green) in Dox-induced, but not uninduced cells (**top** panel); and DAPI-stained (blue) BrdU-positive cells (red; **bottom** panels) at 24 h after release from sterum starvation. (**B**) Quantification of BrdU positivity (percent (%) ratio of BrdU positive to DAPI stained cells). Fluorescent cells were imaged and counted using Image J. Results shown are the means ± SEM of three replicates of a representative experiment; essentially the same results were obtained in three independent experiments. *p* < 0.05 was considered significant; ** represents *p* < 0.01.

The role of CD133 in cell proliferation via AREG upregulation was additionally assessed by immunofluorescent quantification of bromodeoxyuridine (BrdU) incorporation into newly synthesized DNA. Incorporation of BrdU into nascent DNA measures DNA replication in cells as they are stimulated to reenter the cell cycle by serum addition. Cells were induced with Dox for 24 h, plated in serum-free medium for 48 h to synchronize cells in the cell cycle, released into the S-phase, and stimulated to proliferate by the addition of 10% serum. After further incubation for 24 h, cells were pulsed with BrdU and then fixed for immunofluorescence imaging using antibodies specific for BrdU and CD133. CD133 was undetectable in uninduced cells (-Dox), with few nuclei showing BrdU-positivity at 24 h. Consistent with an increased percentage of cells in the S-phase, Dox induction of CD133 was confirmed by immunofluorescent staining (Figure 7, top panels; green fluorescence), coincident with markedly increased numbers of nuclei staining positive for BrdU (Figure 7, lower panels; red fluorescence), compared to uninduced cells. Quantification of the ratios of BrdU- to DAPI-stained cells verified that BrdU-positivity was significantly higher in Dox-induced CD133-expressing cells compared to uninduced controls.

### 3.8. Immunoblot Analysis Reveal Activation of EGFR and the MAPK Pathway in BAKP Cells, as Well as Increased Levels of the Proliferating Cell Nuclear Antigen (PCNA), after Dox-Induction of CD133 Expression

The ERK1/2 module of the MAPK pathway plays a central role in the control of cell proliferation and is activated by growth factors such as EGF. The pathway is initiated by activated RAS protein, which binds to and phosphorylates MEK, which then phosphorylates and activates ERK 1/2, a transcription factor that moves to the nucleus to upregulate expression of multiple genes [31]. Sustained ERK1/2 activation is crucial for efficient G1- to-S-phase progression. ERK1/2 also phosphorylates RSK kinase, which then phosphorylates and inactivates the pro-apoptotic protein BAD, thus increasing cell survival. 

**Figure 8:**
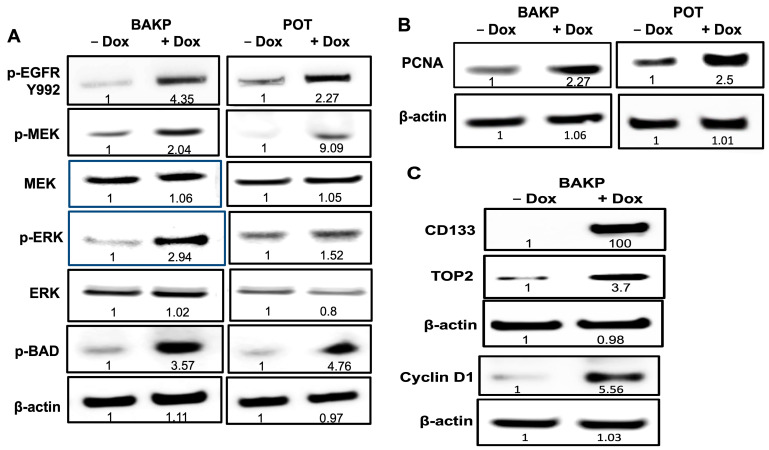
Two melanoma cell lines BAKP and POT, exhibit higher levels of phosphorylation and activation of EGFR and members of the MAPK pathway (p-MEK, p-ERK, p-BAD compared to total MEK, ERK, BAD) (**A**) as well as the cell proliferation marker PCNA and cyclin D1 (**B**) in Dox-induced vs. uninduced cells. (**A**) Cell lysates of BAKP and POT cells incubated with or without 1 µg/mL Dox for 24 h were subjected to immunoblot analysis with antibodies to pEGFR. Immunoblots were stripped of antibodies and re-probed with antibodies to p-MEK, MEK, p-ERK, ERK, p-BAD, and β-actin for loading control. (**B**) Cell lysates of BAKP and POT cells incubated with or without 1 µg/mL Dox for 24 h were subjected to immunoblot analysis with antibodies to the proliferation marker PCNA, then β-actin for loading control. (**C**) BAKP cells incubated with or without 1 µg/mL Dox for 24 h were subjected to Western blot analysis with antibodies to CD133, then stripped of antibodies and re-probed with antibodies to TOP2 and β-actin, as loading control. Immunoblots loaded with the same lysates were likewise probed with antibodies to Cyclin D1, followed by β-actin. Densitometric analysis is shown in immunoblots, comparing intensities of protein bands relative to that of control −Dox cells, after normalizing to β-actin.

Given that CD133 upregulates AREG, which has been implicated in cell proliferation via its binding to and activation of EGFR, which then activates the MAPK pathway [32], we next examined the phosphorylation and activation of EGFR as well as members of the MAPK pathway in two different melanoma cell lines (BAKP and POT) in response to Dox-induced CD133 expression. Cells were induced or not with Dox for 24 h, and cell lysates were subjected to immunoblot analysis with antibodies specific to phosphorylated forms of EGFR (p-EGFR), MEK (p-MEK), ERK (p-ERK), and BAD (p-BAD) compared to total MEK, ERK, and BAD. As expected, CD133 upregulation of AREG expression resulted in EGFR activation, as shown by a 5- and 2-fold increase in p-EGFR in BAKP and POT cells, respectively, after Dox induction (Figure 8A). Likewise, p-MEK levels were elevated 2- and 9-fold, while p-ERK levels were increased by 3- and 2-fold in +Dox BAKP and POT cells, respectively. These markers of an active MAPK pathway leading to cell proliferation and survival were strongly activated, indicating that upregulation of CD133 led to this response. 

We next assessed expression of PCNA by immunoblot analysis, and found that PCNA was upregulated >2-fold in both BAKP and POT melanoma cell lines upon Dox- inducted CD133 expression (Figure 8B), which is consistent with increased cell proliferation, percentage of cells in the S-phase, and BrdU incorporation in nascent DNA in these cells. PCNA is essential for DNA replication, serving as the DNA clamp and processivity factor for DNA polymerases d and e [33], and is therefore used as a marker to assess cell proliferation as it is rarely expressed in non-proliferating cells. PCNA expression is significantly correlated with cell proliferation since levels reach their peak in S-phase and decline in the M and G2 phases [34]. The PCNA index is used as a successful predictor of local and distant recurrences in patients with primary cutaneous melanoma [35]. 

Similar to PCNA, topoisomerase II (TOP2) is also considered a specific marker for cell proliferation as it is dramatically upregulated during the cell cycle [36].The nuclear enzyme plays an essential role in chromosome condensation, chromatid separation, and the relief of torsional stress during DNA transcription and replication. Western blot analysis with antibodies to TOP2 in BAKP cells reveals a 3.7-fold increase in basal levels after Dox induction of CD133 expression (Figure 8C). Consistent with increased PCNA and TOP2 expression, cyclin D1 protein levels were also markedly enhanced after induction of CD133 expression in both melanoma cell lines (Figure 8C). Activation of the MAPK pathway has been linked to PCNA upregulation, given that activation of the BRAF/MEK/ERK pathway upregulates cyclin D1 [12], resulting in phosphorylation of p-RB [37] and activation of E2F1, which in turn binds to and upregulates the S-phase gene promoters, including those of PCNA [38]. 

### 3.9. Exposure of Dox-Induced BAKP Cells to EGFR Inhibitor Gefitinib Reverses CD133-Induced Stimulation of Cell Growth and Activation of the MAPK Pathway

**Figure 9:**
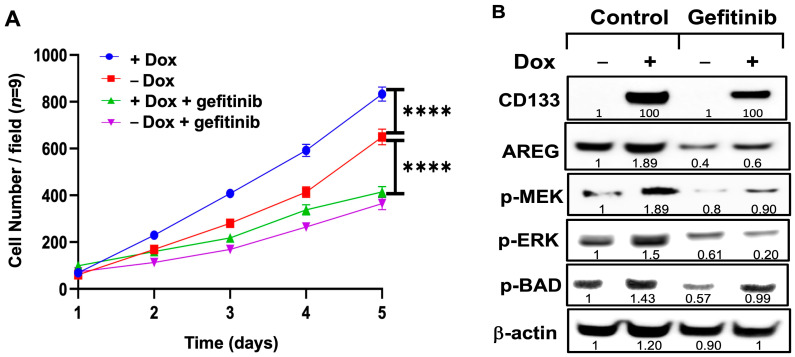
CD133 expression in +Dox cells significantly stimulates cell growth (**A**) and activates the MAPK (p-MEK and p-ERK) and p-BAD pathways, through upregulation of AREG (**B**). This is reversed by the EGFR inhibitor gefitinib, which suppresses cell growth as well as activation of the MAPK pathway. Cells were seeded in equal densities, treated with or without 1 µg/mL Dox for 24 h, and incubated or not with 10 µM gefitinib. (**A**) For cell growth assays, GFP-expressing BAKP cells were seeded in equal numbers in 6-well plates, and then imaged and counted daily for 5 days in triplicate wells, in 3 random microscope fields per well (*n* = 9). Cell growth curves are shown and compared between treatment groups. Results shown are the means ± SEM of three replicates of a representative experiment; essentially the same results were obtained in three independent experiments. *p* < 0.05 was considered significant; **** represents *p* < 0.0001. (**B**) Cell extracts were subjected to immunoblot analysis with antibodies to AREG. Immunoblots were then stripped and re-probed with antibodies to activated forms of members of the MAPK pathway (p-MEK, p-ERK), p-BAD, and β-actin for loading control. Densitometric analysis is shown in immunoblots, comparing intensities of protein bands relative to bands with the highest intensity, after normalizing to β-actin.

While our results showed that CD133 activates an EGFR/MAPK pathway in the two melanoma cell lines tested, the activity of MAPK could alternatively be separate and independent of EGFR activation. We therefore induced BAK or POT cells with Dox in the presence or absence of the EGFR inhibitor, gefitinib. GFP-expressing BAKP cells were seeded at equal densities and induced or not with Dox (1 µg/mL) for 24 h; cells were then further incubated with or without 10 µM gefitinib. Cells were imaged in 3 random microscope fields per well (*n* = 9) with an Olympus immunofluorescence microscope, and counted daily for 5 days to obtain cell growth curves. As expected, Dox induction of CD133 significantly enhanced the rate of cell growth, whereas gefitinib suppressed this increase (Figure 9A). To determine if the CD133-induced activation of the MAPK pathway was also inhibited by gefitinib, cell extracts were derived 24 h after the addition of Dox and/or gefitinib, and subjected to immunoblot analysis. Dox-induced CD133 upregulated AREG, subsequently increasing the activating phosphorylation of MEK, ERK, and BAD. This increase in phosphorylation/activation of the members of the MAPK pathway was markedly inhibited by gefitinib indicating a vital role for EGFR in this pathway.

### 3.10. siRNA Knockdown of AREG Reverses the CD133-Induced Stimulation of Cell Proliferation in BAKP Melanoma Cells

**Figure 10:**
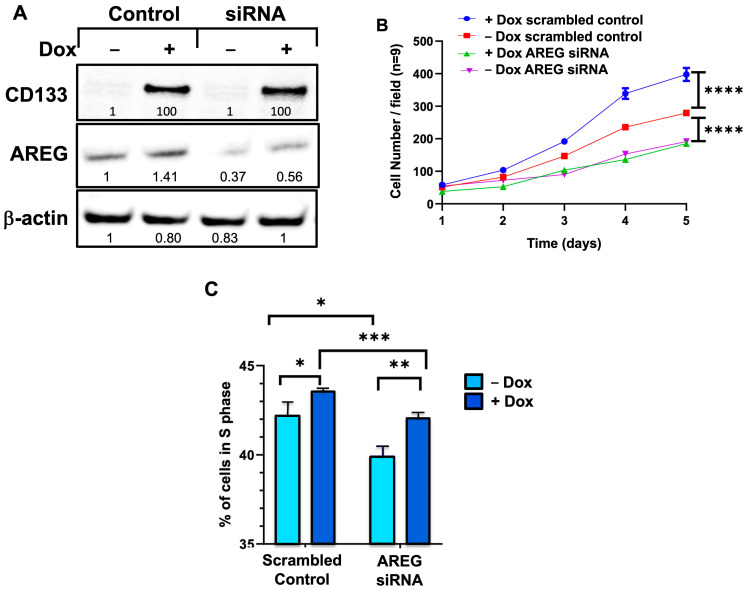
siRNA knockdown of AREG expression in BAKP cells as confirmed by immunoblot analysis (**A**), reverses the CD133-induced stimulation of cell growth (**B**) and increased percentage of cells in S-phase. Cells were seeded in equal densities, treated with or without 1 µg/mL Dox for 24 h, and incubated with scrambled control or AREG siRNA. (**A**) Cell extracts were derived and subjected to immunoblot analysis with antibodies to CD133. Immunoblots were stripped and re-probed with antibodies to AREG, and β-actin for loading control. Densitometric analysis is shown in immunoblots, comparing intensities of protein bands relative to bands with the highest intensity, after normalizing to β-actin. (**B**) GFP-expressing BAKP cells were seeded in equal numbers in 6-well plates +/−Dox, and then imaged and counted daily for 5 days, as described in Materials and Methods (*n* = 9). Cell growth curves are shown and compared between treatment groups. (**C**) Cells were collected at indicated times, fixed in 95% ethanol, stained with PI, and the percentage (%) of cells in S-phase was quantified by flow cytometry. Results shown are the means ± SEM of three replicates of a representative experiment; essentially the same results were obtained in three independent experiments. *p* < 0.05 was considered significant; *, **, ***, **** represent *p* < 0.05, *p* < 0.01, *p* < 0.001, and *p* < 0.0001, respectively.

To determine whether AREG expression is merely correlated with or essential for CD133-induced stimulation of cell proliferation, siRNA directed against *AREG* mRNA was used to knockdown *AREG* expression. Immunoblot analysis verified a 2-fold reduction of immunodetectable AREG in Dox-induced siRNA-knockdown BAKP cells compared to scrambled controls. *AREG* knockdown in BAKP cells effectively reduced AREG protein levels by ~50% (Figure 10A). Cell growth assays were then performed to examine the effects of CD133 induction by Dox as well as the effects of *AREG* knockdown. GFP-expressing BAKP cells were seeded at equal densities and induced with Dox (1 µg/mL) for 24 h; cells were then imaged with an Olympus immunofluorescence microscope for 5 days and counted in three random microscope fields per well (*n* = 9). Finally, the same experiment was performed, and the percentage of cells in the S-phase was assessed by flow cytometry. Whereas upregulation of CD133 by Dox significantly increased cell growth (Figure 10B) and percentage of cells in the S-phase (Figure 10C), siRNA knockdown of AREG expression reversed these effects, indicating that AREG mediates the CD133-induced stimulation of cell growth and proliferation.

## 4. Discussion

The expression profiles of melanoma cells show that CD133 inhibits differentiation and promotes proliferation. The suppression of differentiation manifested as a marked reduction in the expression of enzymes required for melanin pigmentation, including TYRP1 and TYRP2 (Figure 2). Likewise, upregulation of the NF-κB (Figure 3) and EGFR pathways (Figure 8) is consistent with a less differentiated state, as previously described [39]. Further, an increased proliferative state is consistent with previous in vivo studies showing that CD133 significantly increased BAKP xenograft tumor growth in immunocompromised mice [40].

At the single gene level, AREG was determined by RNA-seq to be the most significantly upregulated gene after CD133 (Figure 1). We therefore focused our experiments on determining the roles, if any, of this AREG-dependent pathway. AREG was implicated in cell proliferation, survival, and migration *via* EGFR binding and subsequent MAPK RAS/RAF/MEK/ERK and PI3K/AKT signaling pathways. The current study shows that CD133 upregulates AREG (Figure 2, Figure 4 and Figure 5), consequently increasing activation of EFGR and the MAPK pathway (Figure 8), and enhancing cell proliferation (Figure 6 and Figure 7). Further, this induction of AREG by CD133 was shown in two independent melanoma cell lines, as was its role in the increased MAPK pathway activation and proliferation. To confirm that AREG exerts its activities via EGFR, we blocked the receptor with gefitinib, which prevented downstream biochemical and biological effects (Figure 9). Additionally, the increase in cell growth was AREG-dependent, given that siRNA knockdown of AREG reduced cell proliferation to pre-induction levels (Figure 10). 

*The role(s) of AREG in cancer*. AREG is synthesized as a 252 amino acid precursor transmembrane protein until and if it is cleaved by TACE/ADAM17, which releases the soluble form of AREG [30,41,42]. Both intracellular and secreted forms were found to be upregulated by CD133 in our study (Figure 4 and Figure 5). While AREG plays roles in normal mammary gland growth and differentiation [43], its overexpression is observed in a wide variety of human cancers. In fact, AREG enhances proliferation and invasion of cancer cells of the breast [44,45,46], colon [47], and bladder [48]. A role for AREG in cancer development and progression is also suggested by clinical data, indicating that AREG has prognostic utility in gastric [49], colorectal [50], non-small cell lung [51], and breast cancer [52].

*Potential mechanism of AREG stimulation of proliferation*. Intracellular AREG is a membrane-bound precursor protein that engages in juxtacrine signaling on adjacent cells by binding EGFR and activating major intracellular signaling cascades regulating cell survival, motility, and proliferation. When proteolytically processed by the cell membrane proteases TACE/ADAM17, AREG is secreted, behaves as an autocrine or paracrine factor, and may thus also modify the tumor microenvironment [30]. Both intracellular and secreted AREG function primarily by binding and activating EGFR. While a non-canonical EGFR-independent pathway has also been proposed via nuclear localization of AREG [53], this does not seem to be the case in the current study since we did not observe nuclear AREG (Figure 4D). In other studies, AREG-induced proliferation is mediated through the EGFR, which is exclusively attributable to PI3K signaling, since the MEK inhibitor PD98059 has no effect [54]. We are currently targeting the PI3K pathway using capivasertib (AZD5363), but have not observed any effects on cell proliferation or tumorigenesis unless it is used in combination with the MAPK/MEK inhibitor, trametinib [40]. 

*AREG/EGF feedback loop and use of AREG as a therapeutic target*. AREG autocrine loops are activated in breast and ovarian cancer, and ectopic expression of AREG can induce an AREG/EGFR autocrine loop, rendering T cells EGF-independent [46]. Once established, disruption of this autocrine loop by AREG-neutralizing Ab disrupts survival, proliferation, and invasion [55]. While the importance of an AREG/EGFR loop in melanoma is less studied, a role for AREG expression in melanomagenesis has been demonstrated [53]. Similarly, AREG has been implicated in positive-feedback activation of EGFR in other studies [46,56]. Several mechanisms have been proposed to facilitate the AREG/EGFR feedback loop, including signaling either through ERK/MAPK [32], p38 MAPK [57], or PI3K signaling [58]. The importance of AREG signaling has therefore attracted the attention of investigators looking for therapeutic targets. 

Regardless of the mechanism, these studies illustrate the important contribution of AREG to a key pathway involved in maintaining a malignant phenotype and are therefore an important therapeutic target. Importantly, it was previously shown that AREG-stimulated cell migration and proliferation were largely blocked by the addition of the metalloprotease inhibitor GM6001 to prevent the processing of membrane-bound AREG precursors into soluble AREG in ovarian cancers [46]. The addition of a neutralizing AREG antibody [55] has also been found to block EGFR-stimulated migration, proliferation, and growth of ovarian xenografted tumors. Similarly, downregulation of endogenous AREG by siRNA in the current study prevented the stimulatory effect of AREG on cell proliferation (Figure 10). 

*Limitations and Future Studies.* While transient transfection of the BAKP cells with the scrambled control sequences exerted a slightly cytostatic or cytotoxic effect in cells, which may have impacted the results [59], this was the appropriate necessary control for the AREG siRNA-treated cells, which still exhibited significantly slower cell growth rates compared to the scrambled control cells, indicating a vital role for AREG in cell growth. AREG was shown to be released into the culture medium upon stimulation of CD133/AREG (Figure 4), but the necessity for this cleavage and release of the AREG ligand remains to be clarified.

In any case, these findings point to AREG as a potential therapeutic target in CD133/EGFR-driven melanoma stem cells. While single-agent anti-EGFR therapy may not increase patient survival in all cancer types [60], promising alternative (or adjunct) targeting approaches include regulating AREG release by ADAM-17 inhibition [61], immunological blockade of AREG [55], or disrupting the AREG feedback loop through combined inhibition of YB-1 and mTOR (downstream of PI3K) [62].

## 5. Conclusions

**Figure 11:**
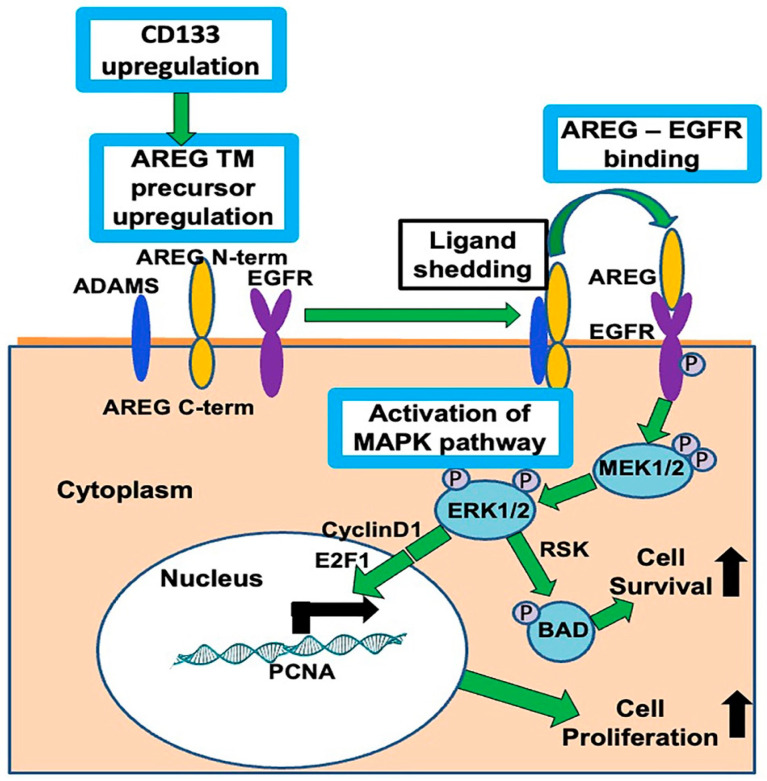
CD133 stimulates cell proliferation and promotes melanoma progression via a novel CD133-AREG-EGFR-MAPK activation pathway in CD133-positive melanoma-initiating stem cells (MICs), summarized as follows: (1) CD133 upregulates AREG, (2) AREG is cleaved to its ligand form by metalloproteinase ADAMS, (3) AREG ligand binds to and activates EGFR, (4) EGFR activates the MAPK pathway (by phosphorylating MEK, which phosphorylates ERK), (5) ERK phosphorylates and inactivates apoptotic protein BAD, leading to increased cell survival, (6) activation of the MEK/ERK pathway upregulates cyclin D1, (7) resulting in activation of E2F1, 8), which in turn binds to and upregulates the S-phase gene promoters, including those of PCNA.

We have shown that induced ectopic expression of CD133 alters the gene expression profile of two independent patient-derived *NRAS* mutant melanoma cell lines. This leads to a less differentiated and more proliferative phenotype, mediated in part by a novel CD133/AREG/EGFR/MAPK pathway (as summarized schematically in Figure 11).

## Data Availability

The original contributions presented in this study are included in the article/Supplementary Material; further inquiries can be dirrected to the corresponding author.

## Supplementary Materials

The following supporting information can be downloaded at: https://www.mdpi.com/article/10.3390/cells13090777/s1, Figure S1: Vector maps and sequences of pLenti-CMV-rtTA3 Blast and pLV-EGFP/Neo-TRE3G-CD133. Figure S2: Full length gels of immunoblots used in all figures.
