# Supplementary material for: CD133 Stimulates Cell Proliferation via the Upregulation of Amphiregulin in Melanoma
Source: Cells. 2024 May 2;13(9):777. doi: 10.3390/cells13090777 (PMC11083289; doi:10.3390/cells13090777)
Supplement: Supplementary file 1 [file cells-13-00777-s001.zip › cells-2925218-Figure S1.pdf]

Simbulan-Rosenthal *etal. Cells 2024*

Supplementary
Figures Figure S1
Vector Maps and Sequences for *pLenti-CMV-rtTA3 Blast and pLV-EGFP/Neo-TRE3G-CD133.*

Vector Summary

**Vector ID**	VB190111-1105mae
**Vector Name**	pLV[TetOn]-EGFP/Neo-TRE3G>hPROM1[NM_001145850.1]
**Vector Size**	11208 bp
**Viral Genome Size**	7733 bp
**Vector Type**	Mammalian Tet-On Inducible Gene Expression Lentiviral Vector
**Inserted Promoter**	TRE3G
**Inserted ORF**	hPROM1[NM_001145850.1]
**Inserted Marker**	EGFP/Neo
**Plasmid Copy Number**	High
**Antibiotic Resistance**	Ampicillin
**Cloning Host**	Stbl3 (or alternative strain)

Note:

Vector Map

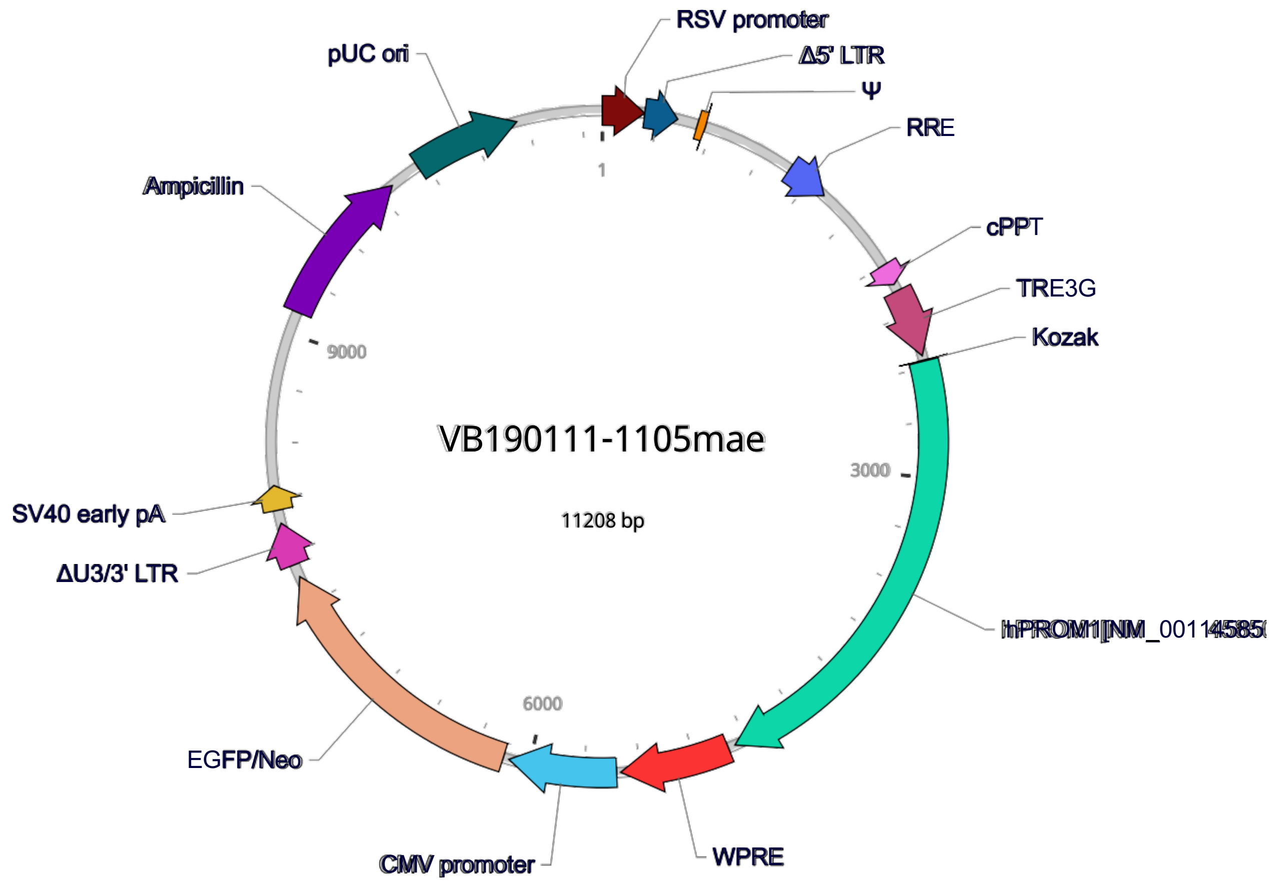

Vector Components

Name	Position	Size (bp)	Type	Description	Application notes
RSV promoter	1-229	229	Promoter	Rous sarcoma virus enhancer/promoter	Strong promoter; drives transcription of viral RNA in packaging cells.
Δ5' LTR	230-410	181	LTR	Truncated HIV-1 5' long terminal repeat	Allows transcription of viral RNA and its packaging into virus.
Ψ	521-565	45	Miscellaneous	Adenovirus packaging signal	Allows packaging of viral DNA into virus.
RRE	1075-1308	234	Miscellaneous	HIV-1 Rev response element	Rev protein binding site that allows Rev-dependent nuclear export of viral RNA during viral packaging.
cPPT	1803-1920	118	Miscellaneous	Central polypurine tract	Facilitates the nuclear import of HIV-1 cDNA through a central DNA flap.
**TRE3G**	1959-2334	376	Promoter	Tetracycline-responsive element promoter (3rd generation)	Bound by transactivator Tet3G in the presence of tetracycline or its analogs (e.g. doxycycline); low background activity.
Kozak	2359-2364	6	Miscellaneous	Kozak translation initiation sequence	Facilitates translation initiation of ATG start codon downstream of the Kozak sequence.
**hPROM1[NM_001145850.1]**	2365-4869	2505	CDS	*None*	*None*
WPRE	4908-5505	598	Miscellaneous	Woodchuck hepatitis virus posttranscriptional regulatory element	Enhances virus stability in packaging cells, leading to higher titer of packaged virus; enhances higher expression of transgenes.
CMV promoter	5527-6114	588	Promoter	Human cytomegalovirus immediate early enhancer/promoter	Strong promoter; may have variable strength in some cell types.

Name	Position	Size (bp)	Type	Description	Application notes
**EGFP/Neo**	6146-7657	1512	CDS	EGFP fused with Neo	Allows cells to be visualized by green fluorescence and resistant to geneticin (G418).
ΔU3/3' LTR	7728-7962	235	LTR	Truncated HIV-1 3' long terminal repeat	Allows packaging of viral RNA into virus; self-inactivates the 5' LTR by a copying mechanism during viral genome integration; contains polyadenylation signal for transcription termination.
SV40 early pA	8035-8169	135	PolyA_signal	Simian virus 40 early polyadenylation signal	Allows transcription termination and polyadenylation of mRNA transcribed by Pol II RNA polymerase.
Ampicillin	9123-9983	861	CDS	Ampicillin resistance gene	Allows E. coli to be resistant to ampicillin.
pUC ori	10154-10742	589	Rep_origin	pUC origin of replication	Facilitates plasmid replication in E. coli; regulates high-copy plasmid number (500-700).

Note: Components added by user are listed in **bold red** text.

Vector Sequence

```
  1 AATGTAGTCT TATGCAATAC TCTTGTAGTC TTGCAACATG GTAACGATGA GTTAGCAACA TGCCTTACAA GGAGAGAAAA
 81 AGCACCGTGC ATGCCGATTG GTGGAAGTAA GGTGGTACGA TCGTGCCTTA TTAGGAAGGC AACAGACGGG TCTGACATGG
161 ATTGGACGAA CCACTGAATT GCCGCATTGC AGAGATATTG TATTTAAGTG CCTAGCTCGA TACATAAACG GGTCTCTCTG
241 GTTAGACCAG ATCTGAGCCT GGGAGCTCTC TGGCTAACTA GGGAACCCAC TGCTTAAGCC TCAATAAAGC TTGCCTTGAG
321 TGCTTCAAGT AGTGTGTGCC CGTCTGTTGT GTGACTCTGG TAACTAGAGA TCCCTCAGAC CCTTTTAGTC AGTGTGGAAA
401 ATCTCTAGCA GTGGCGCCCG AACAGGGACT TGAAAGCGAA AGGGAAACCA GAGGAGCTCT CTCGACGCAG GACTCGGCTT
481 GCTGAAGCGC GCACGGCAAG AGGCGAGGGG CGGCGACTGG TGAGTACGCC AAAAATTTTG ACTAGCGGAG GCTAGAAGGA
561 GAGAGATGGG TGCGAGAGCG TCAGTATTAA GCGGGGGAGA ATTAGATCGC GATGGGAAAA AATTCGGTTA AGGCCAGGGG
641 GAAAGAAAAA ATATAAATTA AAACATATAG TATGGGCAAG CAGGGAGCTA GAACGATTCG CAGTTAATCC TGGCCTGTTA
721 GAAACATCAG AAGGCTGTAG ACAAATACTG GGACAGCTAC AACCATCCCT TCAGACAGGA TCAGAAGAAC TTAGATCATT
801 ATATAATACA GTAGCAACCC TCTATTGTGT GCATCAAAGG ATAGAGATAA AAGACACCAA GGAAGCTTTA GACAAGATAG
881 AGGAAGAGCA AAACAAAAGT AAGACCACCG CACAGCAAGC GGCCGCTGAT CTTCAGACCT GGAGGAGGAG ATATGAGGGA
961 CAATTGGAGA AGTGAATTAT ATAAATATAA AGTAGTAAAA ATTGAACCAT TAGGAGTAGC ACCCACCAAG GCAAAGAGAA
```

1041 GAGTGGTGCA GAGAGAAAAA AGAGCAGTGG GAATAGGAGC TTTGTTCCTT GGGTTCTTGG GAGCAGCAGG AAGCACTATG
1121 GGCGCAGCGT CAATGACGCT GACGGTACAG GCCAGACAAT TATTGTCTGG TATAGTGCAG CAGCAGAACA ATTTGCTGAG
1201 GGCTATTGAG GCGCAACAGC ATCTGTTGCA ACTCACAGTC TGGGGCATCA AGCAGCTCCA GGCAAGAATC CTGGCTGTGG
1281 AAAGATACCT AAAGGATCAA CAGCTCCTGG GGATTTGGGG TTGCTCTGGA AAACTCATTT GCACCACTGC TGTGCCTTGG
1361 AATGCTAGTT GGAGTAATAA ATCTCTGGAA CAGATTTGGA ATCACACGAC CTGGATGGAG TGGGACAGAG AAATTAACAA
1441 TTACACAAGC TTAATACACT CCTTAATTGA AGAATCGCAA AACCAGCAAG AAAAGAATGA ACAAGAATTA TTGGAATTAG
1521 ATAAATGGGC AAGTTTGTGG AATTGGTTTA ACATAACAAA TTGGCTGTGG TATATAAAAT TATTCATAAT GATAGTAGGA
1601 GGCTTGGTAG GTTTAAGAAT AGTTTTTGCT GTACTTTCTA TAGTGAATAG AGTTAGGCAG GGATATTCAC CATTATCGTT
1681 TCAGACCCAC CTCCCAACCC CGAGGGGACC CGACAGGCCC GAAGGAATAG AAGAAGAAGG TGGAGAGAGA GACAGAGACA
1761 GATCCATTCG ATTAGTGAAC GGATCTCGAC GGTATCGCTA GCTTTTAAAA GAAAAGGGGG GATTGGGGGG TACAGTGCAG
1841 GGGAAAGAAT AGTAGACATA ATAGCAACAG ACATACAAAC TAAAGAATTA CAAAAACAAA TTACAAAAAT TCAAAATTTT
1921 ACTAGTGATT ATCGGATCAA CTTTGTATAG AAAAGTTGTT TACTCCCTAT CAGTGATAGA GAACGTATGA AGAGTTTACT
2001 CCCTATCAGT GATAGAGAAC GTATGCAGAC TTTACTCCCT ATCAGTGATA GAGAACGTAT AAGGAGTTTA CTCCCTATCA
2081 GTGATAGAGA ACGTATGACC AGTTTACTCC CTATCAGTGA TAGAGAACGT ATCTACAGTT TACTCCCTAT CAGTGATAGA
2161 GAACGTATAT CCAGTTTACT CCCTATCAGT GATAGAGAAC GTATAAGCTT TAGGCGTGTA CGGTGGGCGC CTATAAAAGC
2241 AGAGCTCGTT TAGTGAACCG TCAGATCGCC TGGAGCAATT CCACAACACT TTTGTCTTAT ACCAACTTTC CGTACCACTT
2321 CCTACCCTCG TAAACAAGTT TGTACAAAAA AGCAGGCTGC CACCATGGCC CTCGTACTCG GCTCCCTGTT GCTGCTGGGG
2401 CTGTGCGGGA ACTCCTTTTC AGGAGGGCAG CCTTCATCCA CAGATGCTCC TAAGGCTTGG AATTATGAAT TGCCTGCAAC
2481 AAATTATGAG ACCCAAGACT CCCATAAAGC TGGACCCATT GGCATTCTCT TTGAACTAGT GCATATCTTT CTCTATGTGG
2561 TACAGCCGCG TGATTTCCCA GAAGATACTT TGAGAAAATT CTTACAGAAG GCATATGAAT CCAAAATTGA TTATGACAAG
2641 CCAGAAACTG TAATCTTAGG TCTAAAGATT GTCTACTATG AAGCAGGGAT TATTCTATGC TGTGTCCTGG GGCTGCTGTT
2721 TATTATTCTG ATGCCTCTGG TGGGGTATTT CTTTTGTATG TGTCGTTGCT GTAACAAATG TGGTGGAGAA ATGCACCAGC
2801 GACAGAAGGA AAATGGGCCC TTCCTGAGGA AATGCTTTGC AATCTCCCTG TTGGTGATTT GTATAATAAT AAGCATTGGC
2881 ATCTTCTATG GTTTTGTGGC AAATCACCAG GTAAGAACCC GGATCAAAAG GAGTCGGAAA CTGGCAGATA GCAATTTCAA
2961 GGACTTGCGA ACTCTCTTGA ATGAAACTCC AGAGCAAATC AAATATATAT TGGCCCAGTA CAACACTACC AAGGACAAGG
3041 CGTTCACAGA TCTGAACAGT ATCAATTCAG TGCTAGGAGG CGGAATTCTT GACCGACTGA GACCCAACAT CATCCCTGTT
3121 CTTGATGAGA TTAAGTCCAT GGCAACAGCG ATCAAGGAGA CCAAAGAGGC GTTGGAGAAC ATGAACAGCA CCTTGAAGAG
3201 CTTGCACCAA CAAAGTACAC AGCTTAGCAG CAGTCTGACC AGCGTGAAAA CTAGCCTGCG GTCATCTCTC AATGACCCTC
3281 TGTGCTTGGT GCATCCATCA AGTGAAACCT GCAACAGCAT CAGATTGTCT CTAAGCCAGC TGAATAGCAA CCCTGAACTG
3361 AGGCAGCTTC CACCCGTGGA TGCAGAACTT GACAACGTTA ATAACGTTCT TAGGACAGAT TTGGATGGCC TGGTCCAACA
3441 GGGCTATCAA TCCCTTAATG ATATACCTGA CAGAGTACAA CGCCAAACCA CGACTGTCGT AGCAGGTATC AAAAGGGTCT
3521 TGAATTCCAT TGGTTCAGAT ATCGACAATG TAACTCAGCG TCTTCCTATT CAGGATATAC TCTCAGCATT CTCTGTTTAT
3601 GTTAATAACA CTGAAAGTTA CATCCACAGA AATTTACCTA CATTGGAAGA GTATGATTCA TACTGGTGGC TGGGTGGCCT
3681 GGTCATCTGC TCTCTGCTGA CCCTCATCGT GATTTTTTAC TACCTGGGCT TACTGTGTGG CGTGTGCGGC TATGACAGGC
3761 ATGCCACCCC GACCACCCGA GGCTGTGTCT CCAACACCGG AGGCGTCTTC CTCATGGTTG GAGTTGGATT AAGTTTCCTC
3841 TTTTGCTGGA TATTGATGAT CATTGTGGTT CTTACCTTTG TCTTTGGTGC AAATGTGGAA AAACTGATCT GTGAACCTTA
3921 CACGAGCAAG GAATTATTCC GGGTTTTGGA TACACCCTAC TTACTAAATG AAGACTGGGA ATACTATCTC TCTGGGAAGC
4001 TATTTAATAA ATCAAAAATG AAGCTCACTT TTGAACAAGT TTACAGTGAC TGCAAAAAAA ATAGAGGCAC TTACGGCACT
4081 CTTCACCTGC AGAACAGCTT CAATATCAGT GAACATCTCA ACATTAATGA GCATACTGGA AGCATAAGCA GTGAATTGGA
4161 AAGTCTGAAG GTAAATCTTA ATATCTTTCT GTTGGGTGCA GCAGGAAGAA AAAACCTTCA GGATTTTGCT GCTTGTGGAA
4241 TAGACAGAAT GAATTATGAC AGCTACTTGG CTCAGACTGG TAAATCCCCC GCAGGAGTGA ATCTTTTATC ATTTGCATAT
4321 GATCTAGAAG CAAAAGCAAA CAGTTTGCCC CCAGGAAATT TGAGGAACTC CCTGAAAAGA GATGCACAAA CTATTAAAAC
4401 AATTCACCAG CAACGAGTCC TTCCTATAGA ACAATCACTG AGCACTCTAT ACCAAAGCGT CAAGATACTT CAACGCACAG
4481 GGAATGGATT GTTGGAGAGA GTAACTAGGA TTCTAGCTTC TCTGGATTTT GCTCAGAACT TCATCACAAA CAATACTTCC
4561 TCTGTTATTA TTGAGGAAAC TAAGAAGTAT GGGAGAACAA TAATAGGATA TTTTGAACAT TATCTGCAGT GGATCGAGTT
4641 CTCTATCAGT GAGAAAGTGG CATCGTGCAA ACCTGTGGCC ACCGCTCTAG ATACTGCTGT TGATGTCTTT CTGTGTAGCT
4721 ACATTATCGA CCCCTTGAAT TTGTTTTGGT TTGGCATAGG AAAAGCTACT GTATTTTTAC TTCCGGCTCT AATTTTTGCG
4801 GTAAAACTGG CTAAGTACTA TCGTCGAATG GATTCGGAGG ACGTGTACGA TGACCCATCA CAACATTGAA CCCAGCTTTC

4881 TTGTACAAAG TGGTGATAAT CGAATTCCGA TAATCAACCT CTGGATTACA AAATTTGTGA AAGATTGACT GGTATTCTTA
4961 ACTATGTTGC TCCTTTTACG CTATGTGGAT ACGCTGCTTT AATGCCTTTG TATCATGCTA TTGCTTCCCG TATGGCTTTC
5041 ATTTTCTCCT CCTTGTATAA ATCCTGGTTG CTGTCTCTTT ATGAGGAGTT GTGGCCCGTT GTCAGGCAAC GTGGCGTGGT
5121 GTGCACTGTG TTTGCTGACG CAACCCCCAC TGGTTGGGGC ATTGCCACCA CCTGTCAGCT CCTTTCCGGG ACTTTCGCTT
5201 TCCCCCTCCC TATTGCCACG GCGGAACTCA TCGCCGCCTG CCTTGCCCGC TGCTGGACAG GGGCTCGGCT GTTGGGCACT
5281 GACAATTCCG TGGTGTTGTC GGGGAAGCTG ACGTCCTTTC CATGGCTGCT CGCCTGTGTT GCCACCTGGA TTCTGCGCGG
5361 GACGTCCTTC TGCTACGTCC CTTCGGCCCT CAATCCAGCG GACCTTCCTT CCCGCGGCCT GCTGCCGGCT CTGCGGCCTC
5441 TTCCGCGTCT TCGCCTTCGC CCTCAGACGA GTCGGATCTC CCTTTGGGCC GCCTCCCCGC ATCGGGAATT CCCGCGGTTC
5521 GAACGCGTTG ACATTGATTA TTGACTAGTT ATTAATAGTA ATCAATTACG GGGTCATTAG TTCATAGCCC ATATATGGAG
5601 TTCCGCGTTA CATAACTTAC GGTAAATGGC CCGCCTGGCT GACCGCCCAA CGACCCCCGC CCATTGACGT CAATAATGAC
5681 GTATGTTCCC ATAGTAACGC CAATAGGGAC TTTCCATTGA CGTCAATGGG TGGAGTATTT ACGGTAAACT GCCCACTTGG
5761 CAGTACATCA AGTGTATCAT ATGCCAAGTA CGCCCCCTAT TGACGTCAAT GACGGTAAAT GGCCCGCCTG GCATTATGCC
5841 CAGTACATGA CCTTATGGGA CTTTCCTACT TGGCAGTACA TCTACGTATT AGTCATCGCT ATTACCATGG TGATGCGGTT
5921 TTGGCAGTAC ATCAATGGGC GTGGATAGCG GTTTGACTCA CGGGGATTTC CAAGTCTCCA CCCCATTGAC GTCAATGGGA
6001 GTTTGTTTTG GCACCAAAAT CAACGGGACT TTCCAAAATG TCGTAACAAC TCCGCCCCAT TGACGCAAAT GGGCGGTAGG
6081 CGTGTACGGT GGGAGGTCTA TATAAGCAGA GCTCTCTGGC TAACTAGAGA ACCCACTGCG CCACCATGGT GAGCAAGGGC
6161 GAGGAGCTGT TCACCGGGGT GGTGCCCATC CTGGTCGAGC TGGACGGCGA CGTAAACGGC CACAAGTTCA GCGTGTCCGG
6241 CGAGGGCGAG GGCGATGCCA CCTACGGCAA GCTGACCCTG AAGTTCATCT GCACCACCGG CAAGCTGCCC GTGCCCTGGC
6321 CCACCCTCGT GACCACCCTG ACCTACGGCG TGCAGTGCTT CAGCCGCTAC CCCGACCACA TGAAGCAGCA CGACTTCTTC
6401 AAGTCCGCCA TGCCCGAAGG CTACGTCCAG GAGCGCACCA TCTTCTTCAA GGACGACGGC AACTACAAGA CCCGCGCCGA
6481 GGTGAAGTTC GAGGGCGACA CCCTGGTGAA CCGCATCGAG CTGAAGGGCA TCGACTTCAA GGAGGACGGC AACATCCTGG
6561 GGCACAAGCT GGAGTACAAC TACAACAGCC ACAACGTCTA TATCATGGCC GACAAGCAGA AGAACGGCAT CAAGGTGAAC
6641 TTCAAGATCC GCCACAACAT CGAGGACGGC AGCGTGCAGC TCGCCGACCA CTACCAGCAG AACACCCCCA TCGGCGACGG
6721 CCCCGTGCTG CTGCCCGACA ACCACTACCT GAGCACCCAG TCCGCCCTGA GCAAAGACCC CAACGAGAAG CGCGATCACA
6801 TGGTCCTGCT GGAGTTCGTG ACCGCCGCCG GGATCACTCT CGGCATGGAC GAGCTGTACA AGATGATTGA ACAAGATGGA
6881 TTGCACGCAG GTTCTCCGGC CGCTTGGGTG GAGAGGCTAT TCGGCTATGA CTGGGCACAA CAGACAATCG GCTGCTCTGA
6961 TGCCGCCGTG TTCCGGCTGT CAGCGCAGGG GCGCCCGGTT CTTTTTGTCA AGACCGACCT GTCCGGTGCC CTGAATGAAC
7041 TGCAAGACGA GGCAGCGCGG CTATCGTGGC TGGCCACGAC GGGCGTTCCT TGCGCAGCTG TGCTCGACGT TGTCACTGAA
7121 GCGGGAAGGG ACTGGCTGCT ATTGGGCGAA GTGCCGGGGC AGGATCTCCT GTCATCTCAC CTTGCTCCTG CCGAGAAAGT
7201 ATCCATCATG GCTGATGCAA TGCGGCGGCT GCATACGCTT GATCCGGCTA CCTGCCCATT CGACCACCAA GCGAAACATC
7281 GCATCGAGCG AGCACGTACT CGGATGGAAG CCGGTCTTGT CGATCAGGAT GATCTGGACG AAGAGCATCA GGGGCTCGCG
7361 CCAGCCGAAC TGTTCGCCAG GCTCAAGGCG AGCATGCCCG ACGGCGAGGA TCTCGTCGTG ACCCATGGCG ATGCCTGCTT
7441 GCCGAATATC ATGGTGGAAA ATGGCCGCTT TTCTGGATTC ATCGACTGTG GCCGGCTGGG TGTGGCGGAC CGCTATCAGG
7521 ACATAGCGTT GGCTACCCGT GATATTGCTG AAGAGCTTGG CGGCGAATGG GCTGACCGCT TCCTCGTGCT TTACGGTATC
7601 GCCGCTCCCG ATTCGCAGCG CATCGCCTTC TATCGCCTTC TTGACGAGTT CTTCTGAGGT ACCTTTAAGA CCAATGACTT
7681 ACAAGGCAGC TGTAGATCTT AGCCACTTTT TAAAAGAAAA GGGGGGACTG GAAGGGCTAA TTCACTCCCA ACGAAGACAA
7761 GATCTGCTTT TTGCTTGTAC TGGGTCTCTC TGGTTAGACC AGATCTGAGC CTGGGAGCTC TCTGGCTAAC TAGGGAACCC
7841 ACTGCTTAAG CCTCAATAAA GCTTGCCTTG AGTGCTTCAA GTAGTGTGTG CCCGTCTGTT GTGTGACTCT GGTAACTAGA
7921 GATCCCTCAG ACCCTTTTAG TCAGTGTGGA AAATCTCTAG CAGTAGTAGT TCATGTCATC TTATTATTCA GTATTTATAA
8001 CTTGCAAAGA AATGAATATC AGAGAGTGAG AGGAACTTGT TTATTGCAGC TTATAATGGT TACAAATAAA GCAATAGCAT
8081 CACAAATTTC ACAAATAAAG CATTTTTTTC ACTGCATTCT AGTTGTGGTT TGTCCAAACT CATCAATGTA TCTTATCATG
8161 TCTGGCTCTA GCTATCCCGC CCCTAACTCC GCCCATCCCG CCCCTAACTC CGCCCAGTTC CGCCCATTCT CCGCCCCATG
8241 GCTGACTAAT TTTTTTTATT TATGCAGAGG CCGAGGCCGC CTCGGCCTCT GAGCTATTCC AGAAGTAGTG AGGAGGCTTT
8321 TTTGGAGGCC TAGGGACGTA CCCAATTCGC CCTATAGTGA GTCGTATTAC GCGCGCTCAC TGGCCGTCGT TTTACAACGT
8401 CGTGACTGGG AAAACCCTGG CGTTACCCAA CTTAATCGCC TTGCAGCACA TCCCCCTTTC GCCAGCTGGC GTAATAGCGA
8481 AGAGGCCCGC ACCGATCGCC CTTCCCAACA GTTGCGCAGC CTGAATGGCG AATGGGACGC GCCCTGTAGC GGCGCATTAA
8561 GCGCGGCGGG TGTGGTGGTT ACGCGCAGCG TGACCGCTAC ACTTGCCAGC GCCCTAGCGC CCGCTCCTTT CGCTTTCTTC
8641 CCTTCCTTTC TCGCCACGTT CGCCGGCTTT CCCCGTCAAG CTCTAAATCG GGGGCTCCCT TTAGGGTTCC GATTTAGTGC

8721 TTTACGGCAC CTCGACCCCA AAAAACTTGA TTAGGGTGAT GGTTCACGTA GTGGGCCATC GCCCTGATAG ACGGTTTTTC
8801 GCCCTTTGAC GTTGGAGTCC ACGTTCTTTA ATAGTGGACT CTTGTTCCAA ACTGGAACAA CACTCAACCC TATCTCGGTC
8881 TATTCTTTTG ATTTATAAGG GATTTTGCCG ATTTCGGCCT ATTGGTTAAA AAATGAGCTG ATTTAACAAA AATTTAACGC
8961 GAATTTTAAC AAAATATTAA CGCTTACAAT TTAGGTGGCA CTTTTCGGGG AAATGTGCGC GGAACCCCTA TTTGTTTATT
9041 TTTCTAAATA CATTCAAATA TGTATCCGCT CATGAGACAA TAACCCTGAT AAATGCTTCA ATAATATTGA AAAAGGAAGA
9121 GTATGAGTAT TCAACATTTC CGTGTCGCCC TTATTCCCTT TTTTGCGGCA TTTTGCCTTC CTGTTTTTGC TCACCCAGAA
9201 ACGCTGGTGA AAGTAAAAGA TGCTGAAGAT CAGTTGGGTG CACGAGTGGG TTACATCGAA CTGGATCTCA ACAGCGGTAA
9281 GATCCTTGAG AGTTTTCGCC CCGAAGAACG TTTTCCAATG ATGAGCACTT TTAAAGTTCT GCTATGTGGC GCGGTATTAT
9361 CCCGTATTGA CGCCGGGCAA GAGCAACTCG GTCGCCGCAT ACACTATTCT CAGAATGACT TGGTTGAGTA CTCACCAGTC
9441 ACAGAAAAGC ATCTTACGGA TGGCATGACA GTAAGAGAAT TATGCAGTGC TGCCATAACC ATGAGTGATA ACACTGCGGC
9521 CAACTTACTT CTGACAACGA TCGGAGGACC GAAGGAGCTA ACCGCTTTTT TGCACAACAT GGGGGATCAT GTAACTCGCC
9601 TTGATCGTTG GGAACCGGAG CTGAATGAAG CCATACCAAA CGACGAGCGT GACACCACGA TGCCTGTAGC AATGGCAACA
9681 ACGTTGCGCA AACTATTAAC TGGCGAACTA CTTACTCTAG CTTCCCGGCA ACAATTAATA GACTGGATGG AGGCGGATAA
9761 AGTTGCAGGA CCACTTCTGC GCTCGGCCCT TCCGGCTGGC TGGTTTATTG CTGATAAATC TGGAGCCGGT GAGCGTGGGT
9841 CTCGCGGTAT CATTGCAGCA CTGGGGCCAG ATGGTAAGCC CTCCCGTATC GTAGTTATCT ACACGACGGG GAGTCAGGCA
9921 ACTATGGATG AACGAAATAG ACAGATCGCT GAGATAGGTG CCTCACTGAT TAAGCATTGG TAACTGTCAG ACCAAGTTTA
10001 CTCATATATA CTTTAGATTG ATTTAAAACT TCATTTTTAA TTTAAAAGGA TCTAGGTGAA GATCCTTTTT GATAATCTCA
10081 TGACCAAAAT CCCTTAACGT GAGTTTTCGT TCCACTGAGC GTCAGACCCC GTAGAAAAGA TCAAAGGATC TTCTTGAGAT
10161 CCTTTTTTTC TGCGCGTAAT CTGCTGCTTG CAAACAAAAA AACCACCGCT ACCAGCGGTG GTTTGTTTGC CGGATCAAGA
10241 GCTACCAACT CTTTTTCCGA AGGTAACTGG CTTCAGCAGA GCGCAGATAC CAAATACTGT TCTTCTAGTG TAGCCGTAGT
10321 TAGGCCACCA CTTCAAGAAC TCTGTAGCAC CGCCTACATA CCTCGCTCTG CTAATCCTGT TACCAGTGGC TGCTGCCAGT
10401 GGCGATAAGT CGTGTCTTAC CGGGTTGGAC TCAAGACGAT AGTTACCGGA TAAGGCGCAG CGGTCGGGCT GAACGGGGGG
10481 TTCGTGCACA CAGCCCAGCT TGGAGCGAAC GACCTACACC GAACTGAGAT ACCTACAGCG TGAGCTATGA GAAAGCGCCA
10561 CGCTTCCCGA AGAGAGAAAG GCGGACAGGT ATCCGGTAAG CGGCAGGGTC GGAACAGGAG AGCGCACGAG GGAGCTTCCA
10641 GGGGGAAACG CCTGGTATCT TTATAGTCCT GTCGGGTTTC GCCACCTCTG ACTTGAGCGT CGATTTTTGT GATGCTCGTC
10721 AGGGGGGCGG AGCCTATGGA AAAACGCCAG CAACGCGGCC TTTTTACGGT TCCTGGCCTT TTGCTGGCCT TTTGCTCACA
10801 TGTTCTTTCC TGCGTTATCC CCTGATTCTG TGGATAACCG TATTACCGCC TTTGAGTGAG CTGATACCGC TCGCCGCAGC
10881 CGAACGACCG AGCGCAGCGA GTCAGTGAGC GAGGAAGCGG AAGAGCGCCC AATACGCAAA CCGCCTCTCC CCGCGCGTTG
10961 GCCGATTCAT TAATGCAGCT GGCACGACAG GTTTCCCGAC TGGAAAGCGG GCAGTGAGCG CAACGCAATT AATGTGAGTT
11041 AGCTCACTCA TTAGGCACCC CAGGCTTTAC ACTTTATGCT TCCGGCTCGT ATGTTGTGTG GAATTGTGAG CGGATAACAA
11121 TTTCACACAG GAAACAGCTA TGACCATGAT TACGCCAAGC GCGCAATTAA CCCTCACTAA AGGGAACAAA AGCTGGAGCT
11201 GCAAGCTT
