# Supplementary material for: CD133 Stimulates Cell Proliferation via the Upregulation of Amphiregulin in Melanoma
Source: Cells. 2024 May 2;13(9):777. doi: 10.3390/cells13090777 (PMC11083289; doi:10.3390/cells13090777)
Supplement: Supplementary file 1 [file cells-13-00777-s001.zip › cells-2925218-Figure S2.pdf]

Simbulan-Rosenthal *etal. Cells 2024*

Supplementary Figures
Figure S2
Full Gel Images

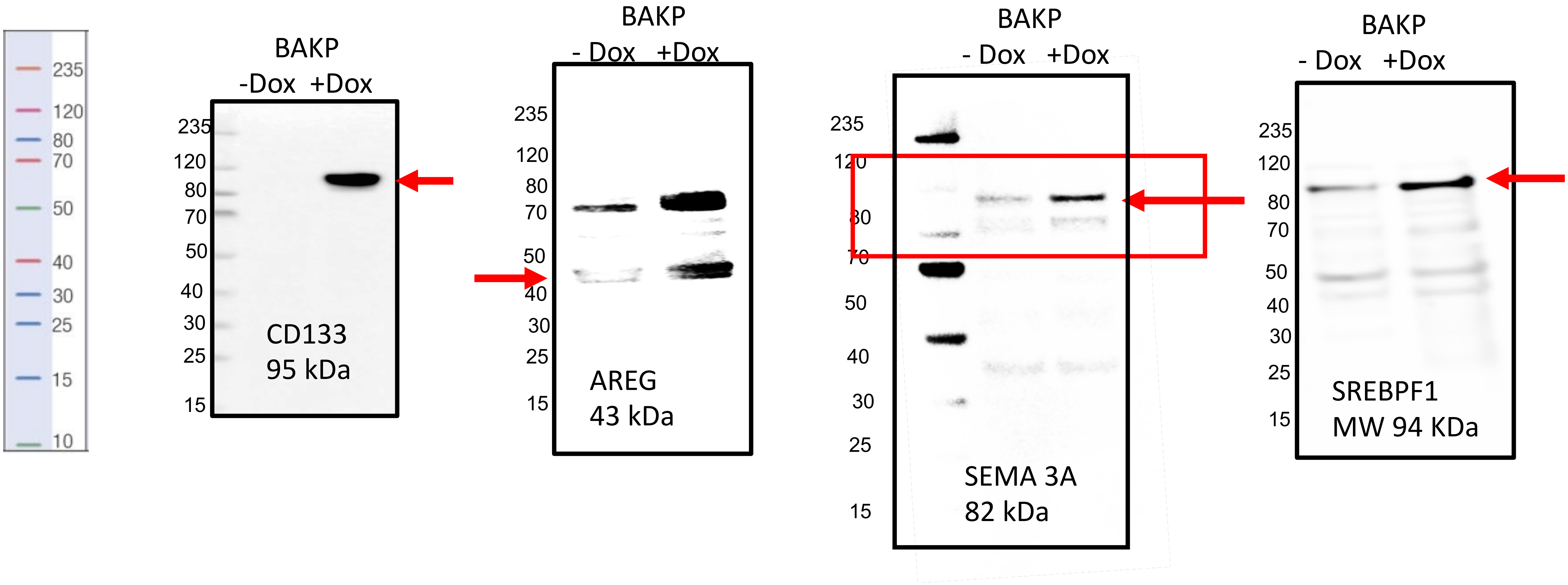

Figure S2a, The original image of Western Blot in Figure 2B.

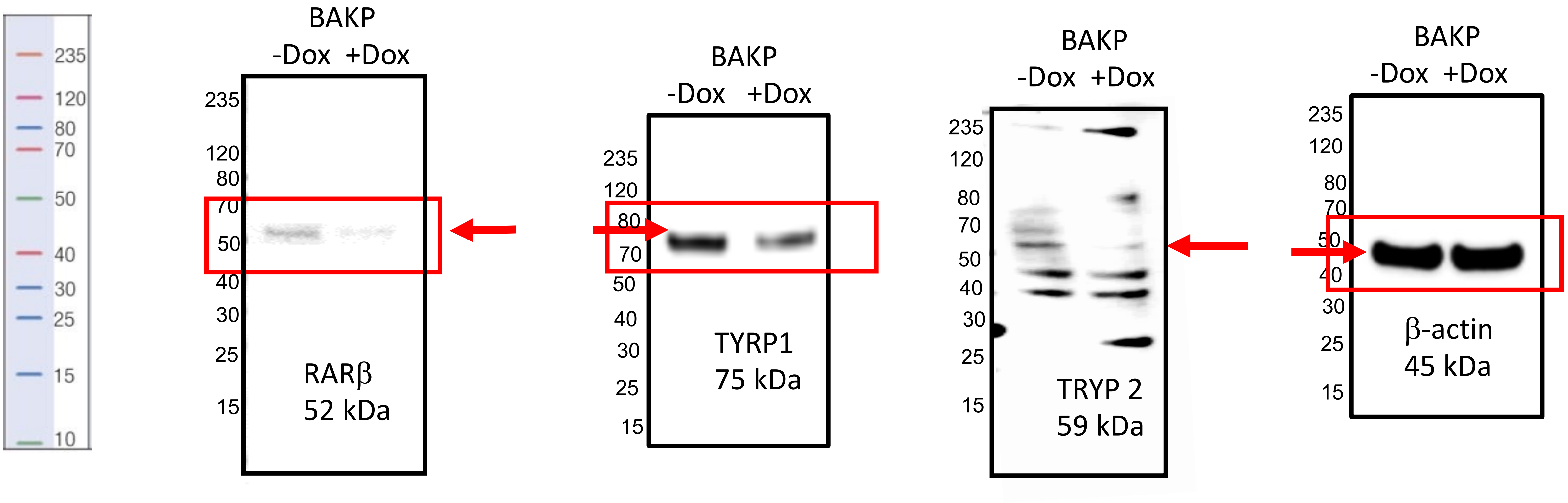

Figure S2a, The original image of Western Blot in Figure 2B.

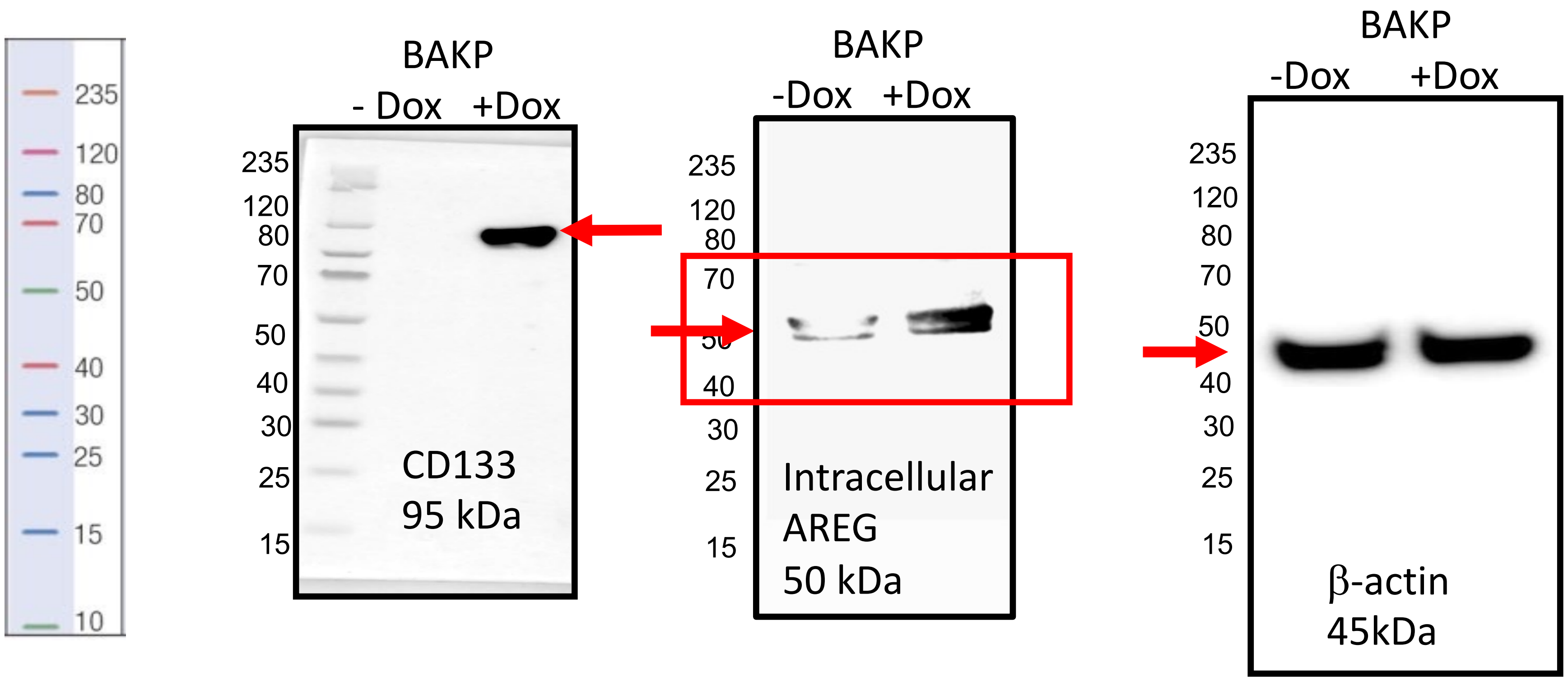

Figure S2b, The original image of Western Blot in Figure 4B.

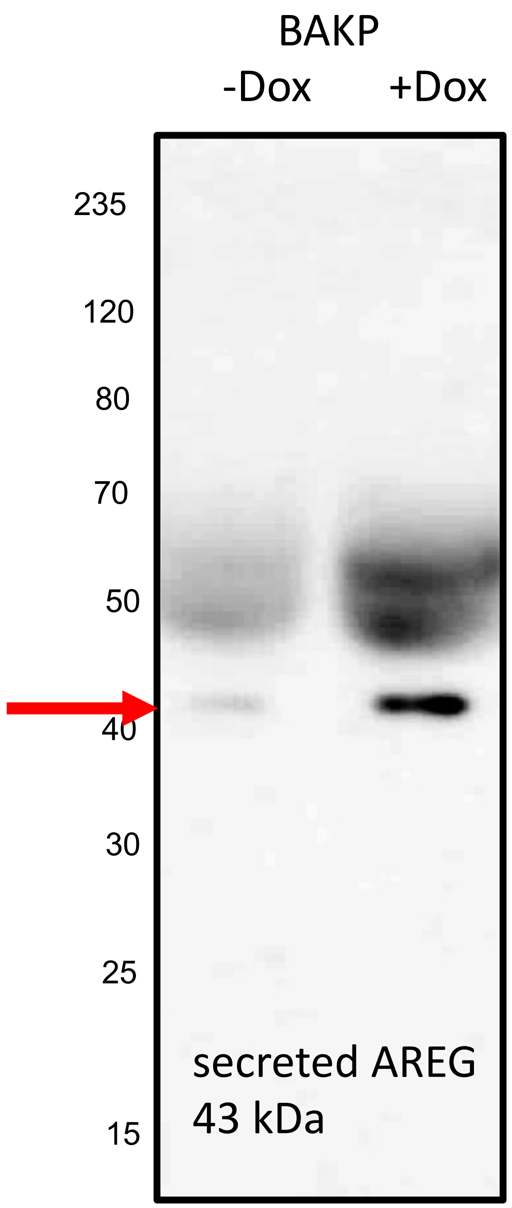

Figure 4C

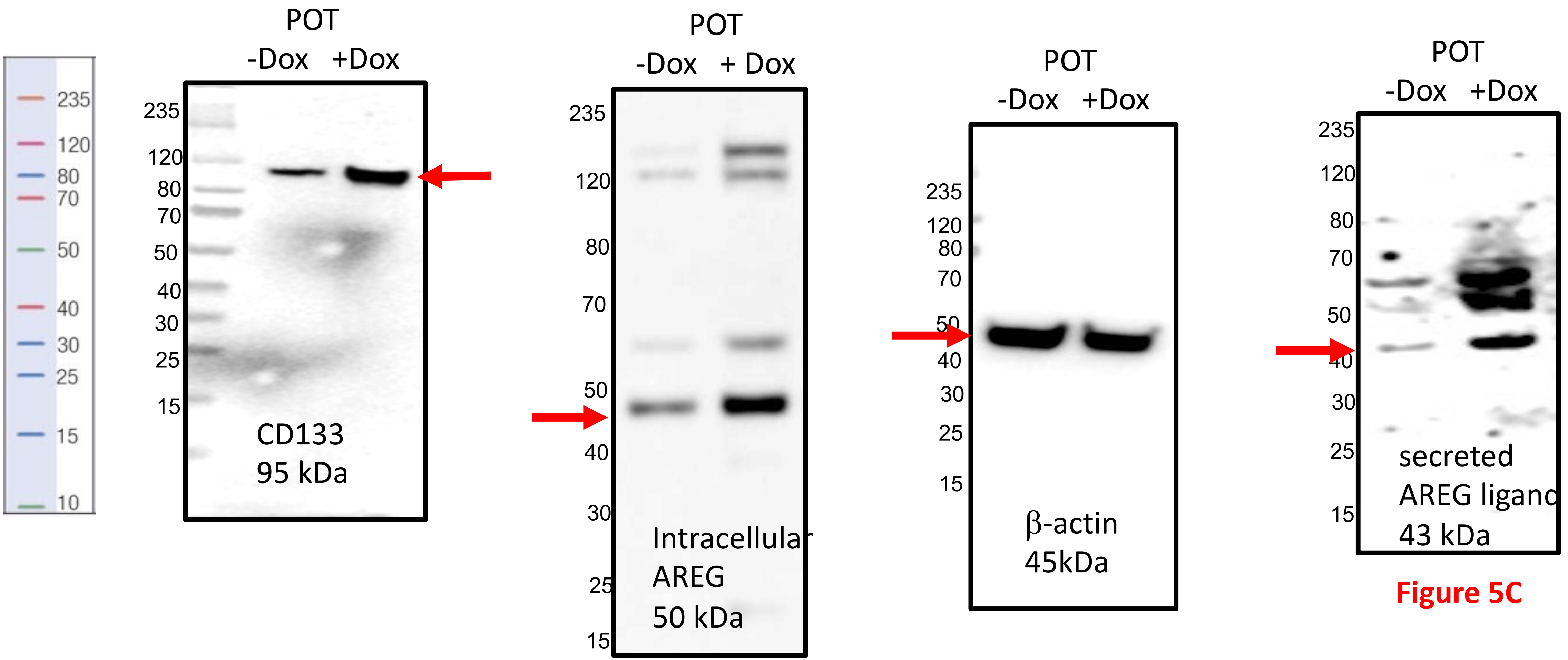

Figure S2c, The original image of Western Blot in Figure 5B, 5C.

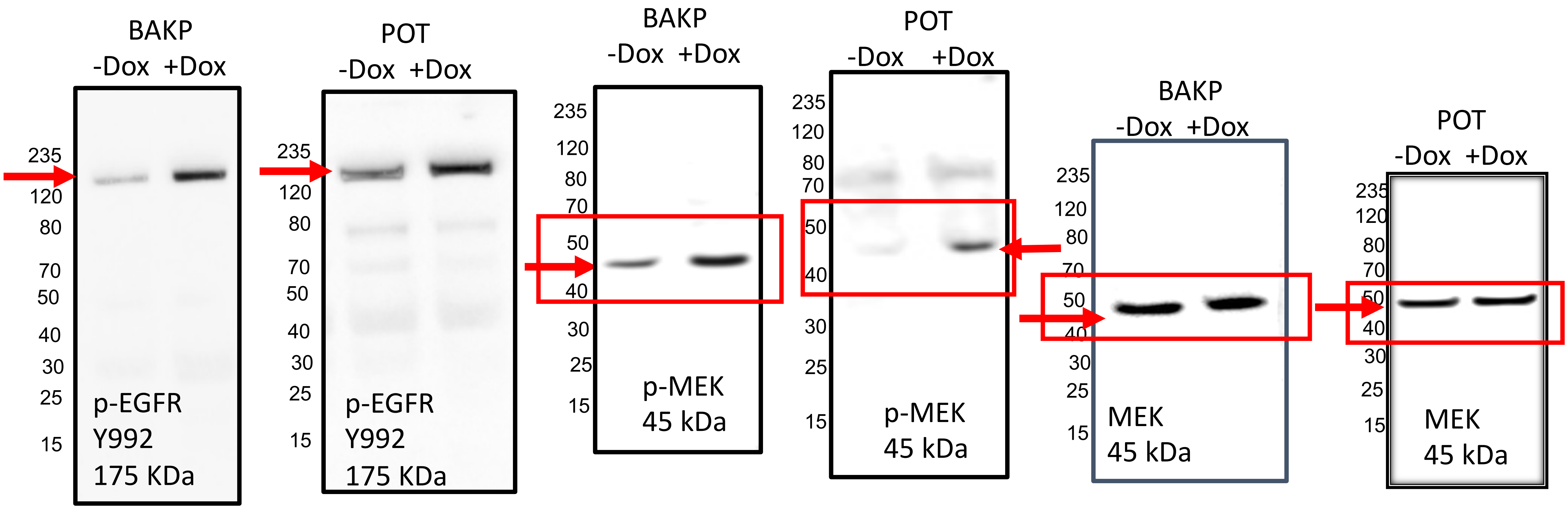

Figure S2d, The original image of Western Blot in Figure 8A.

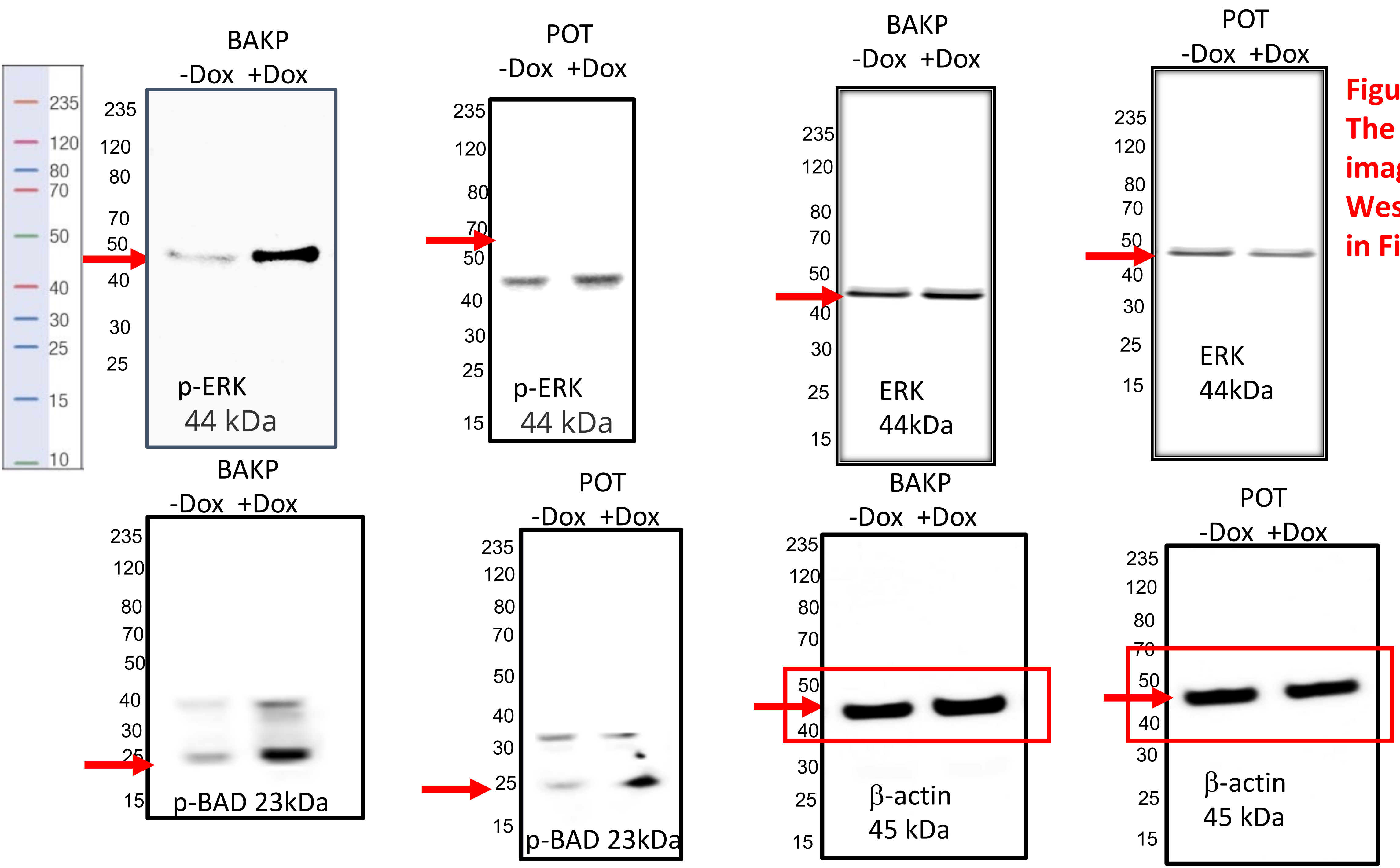

Figure S2d, The original image of Western Blot in Figure 8A.

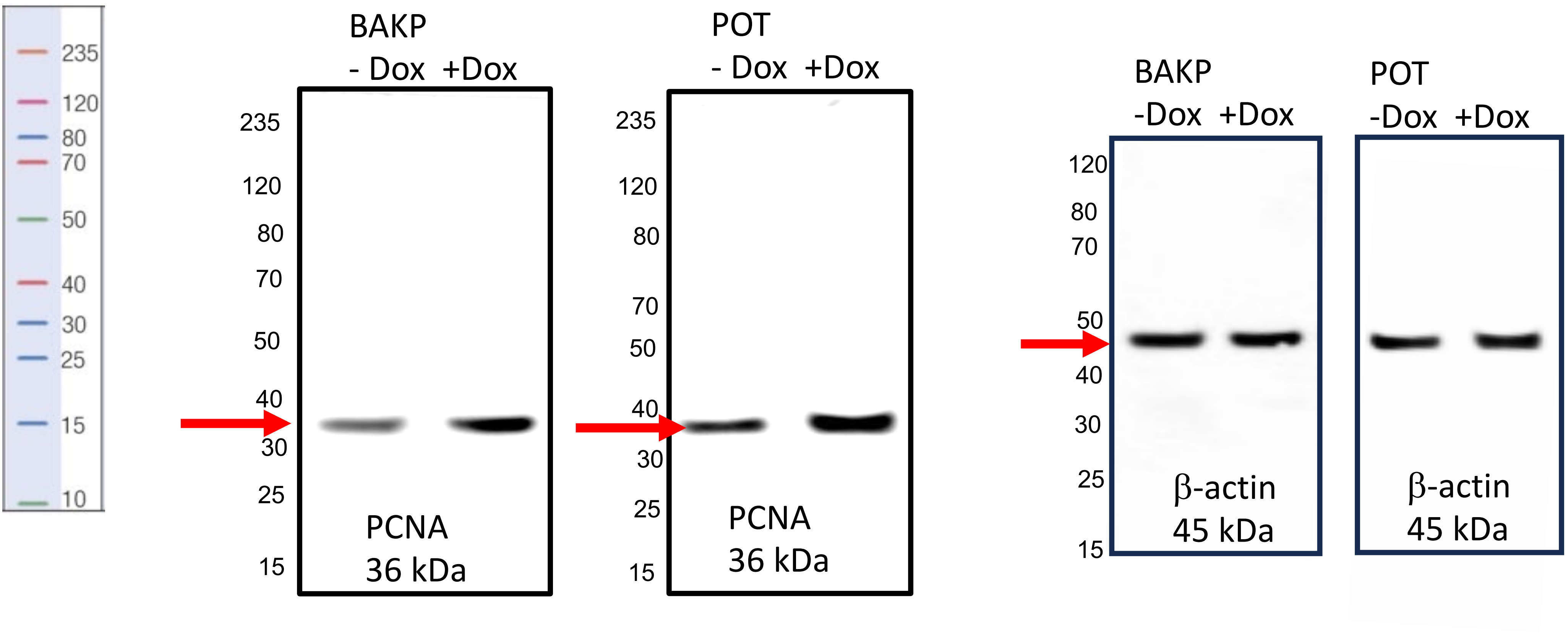

Figure S2e, The original image of Western Blot in Figure 8B.

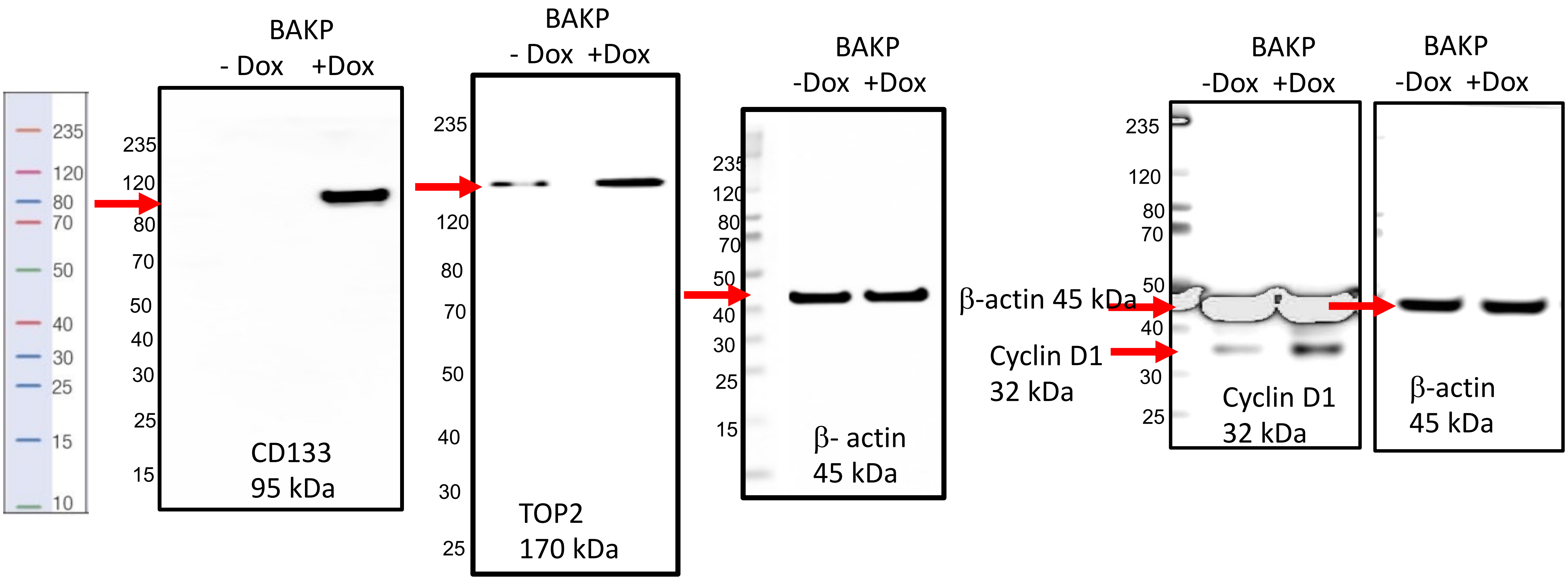
235
120
80
70
50
40
30
25
15
10
BAKP
- Dox +Dox
235
120
80
70
50
40
30
25
15
CD133
95 kDa
BAKP
- Dox +Dox
235
120
80
70
50
40
30
25
TOP2
170 kDa
BAKP
-Dox +Dox
235
120
80
70
50
40
30
25
15
β- actin
45 kDa
β-actin 45 kDa
Cyclin D1
32 kDa
BAKP
-Dox +Dox
235
120
80
70
50
40
30
25
Cyclin D1
32 kDa
BAKP
-Dox +Dox
β-actin
45 kDa

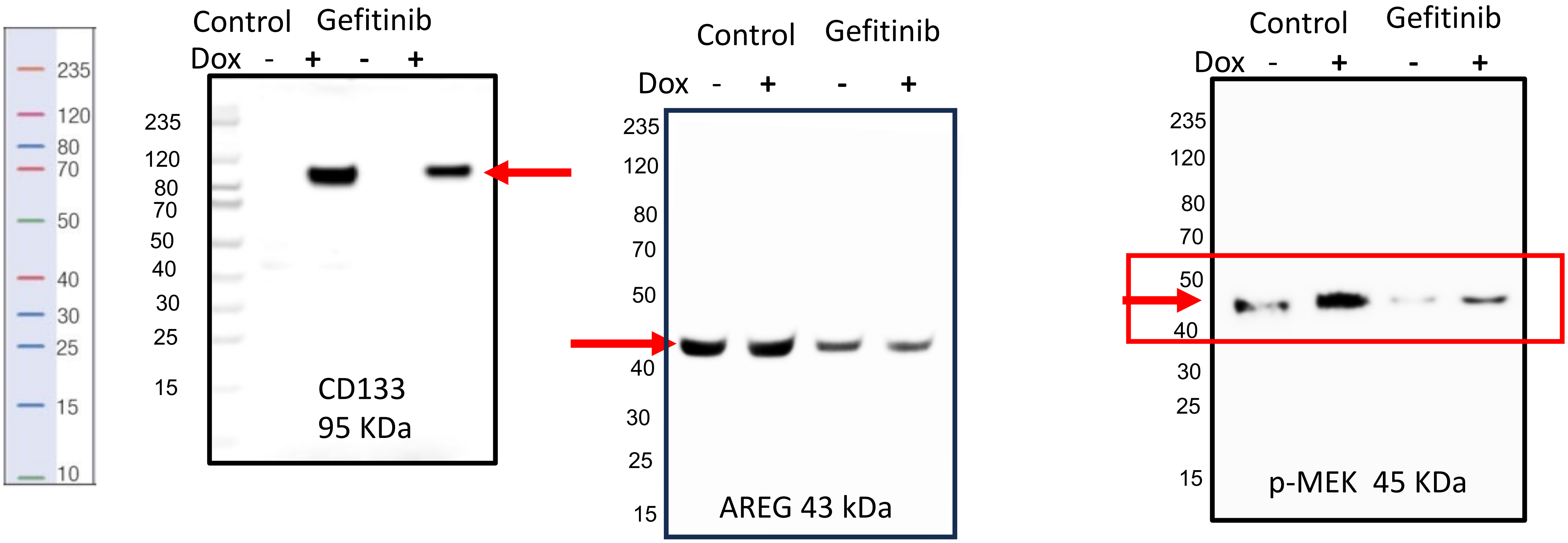

Figure S2f, The original image of Western Blot in Figure 9B.

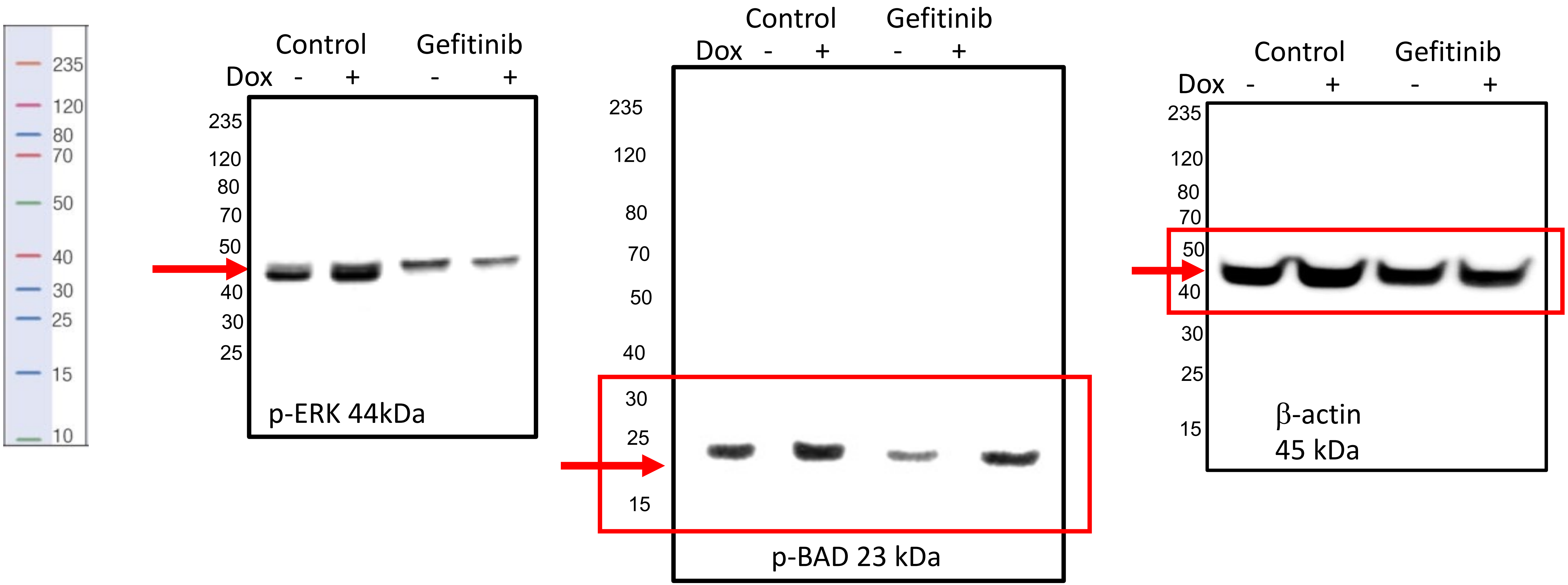

Figure S2f, The original image of Western Blot in Figure 9B.

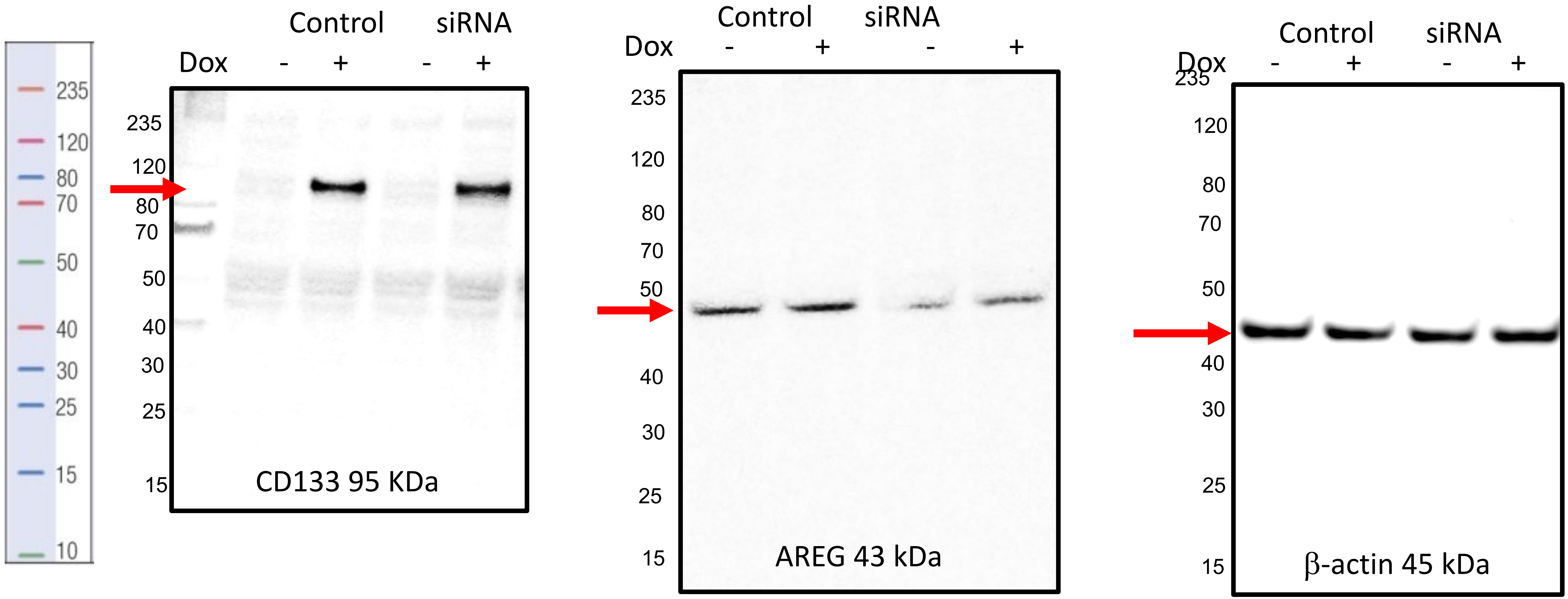

Figure S2g, The original image of Western Blot in Figure 10A.
